# The Use of Amino Sugars by *Bacillus subtilis*: Presence of a Unique Operon for the Catabolism of Glucosamine

**DOI:** 10.1371/journal.pone.0063025

**Published:** 2013-05-08

**Authors:** Isabelle Gaugué, Jacques Oberto, Harald Putzer, Jacqueline Plumbridge

**Affiliations:** 1 CNRS-UPR9073 (affiliated with Université Diderot, Sorbonne Paris Cité), Institut de Biologie Physico-Chimique, Paris, France; 2 CNRS-UMR8621 Institut de Génétique et Microbiologie, Université Paris XI, Orsay, France; University of South Florida College of Medicine, United States of America

## Abstract

*B. subtilis* grows more rapidly using the amino sugar glucosamine as carbon source, than with N-acetylglucosamine. Genes for the transport and metabolism of N-acetylglucosamine (*nagP* and *nagAB*) are found in all the sequenced *Bacilli* (except *Anoxybacillus flavithermus*). In *B. subtilis* there is an additional operon (*gamAP*) encoding second copies of genes for the transport and catabolism of glucosamine. We have developed a method to make multiple deletion mutations in *B. subtilis* employing an excisable spectinomycin resistance cassette. Using this method we have analysed the contribution of the different genes of the *nag* and *gam* operons for their role in utilization of glucosamine and N-acetylglucosamine. Faster growth on glucosamine is due to the presence of the *gamAP* operon, which is strongly induced by glucosamine. Although the *gamA* and *nagB* genes encode isozymes of GlcN6P deaminase, catabolism of N-acetylglucosamine relies mostly upon the *gamA* gene product. The genes for use of N-acetylglucosamine, *nagAB* and *nagP,* are repressed by YvoA (NagR), a GntR family regulator, whose gene is part of the *nagAB yvoA(nagR)* operon. The *gamAP* operon is repressed by YbgA, another GntR family repressor, whose gene is expressed divergently from *gamAP*. The *nagAB yvoA* synton is found throughout the *Bacilli* and most firmicutes. On the other hand the *ybgA-gamAP* synton, which includes the *ybgB* gene for a small protein of unknown provenance, is only found in *B. subtilis* (and a few very close relatives). The origin of *ybgBA-gamAP* grouping is unknown but synteny analysis suggests lateral transfer from an unidentified donor. The presence of *gamAP* has enabled *B. subtilis* to efficiently use glucosamine as carbon source.

## Introduction

**Figure 1:**
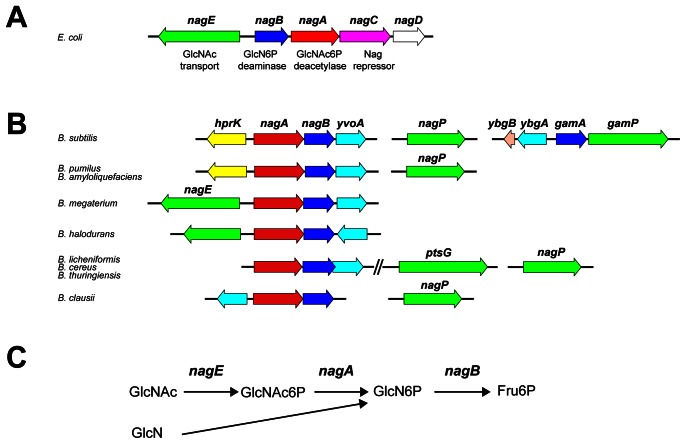
Genes for the metabolism of GlcN and GlcNAc. A. The *nag* operon in *E. coli.* B. The *nag/gam* operons in *B. subtilis* and representative *Bacillaceae.* Genes encoding homologous proteins in the different genomes are shown by similar shading: GlcN6P deaminase, dark blue; GlcNAc6P deacetylase, red; PTS transporter, green; GntR type regulators, cyan; HPR kinase, yellow; NagC (ROK family) regulator, magenta. The *yvoA* gene in *B. subtilis* has been renamed *nagR* [9]. C. Metabolism of GlcNAc and GlcN. Transport of GlcN or GlcNAc by the PTS produces GlcN6P and GlcNAc6P. GlcNAc6P is deacetylated to give GlcN6P, which is deaminated to fructose-6P. *E. coli* gene names are shown, in *E. coli* glucosamine is transported by the *manXYZ* encoded PTS transporter (not shown).

The rapidly expanding number of sequenced genomes required the development of largely automated annotation pipelines built around individual tools such as FGENESB [1] or IMG ER [2]. Nevertheless the assignment of a gene′s function in newly sequenced organisms relies essentially on homology and synteny. Reizer *et a*l [3] examined the DNA sequence of the *B. subtilis* chromosome to identify genes belonging to the family of phosphotransferase (PTS) transporters and found two genes with high similarity to the *nagE* gene, encoding the N-acetylglucosamine (GlcNAc) specific transporter of *E. coli*. Sugar uptake by the PTS results in its simultaneous phosphorylation producing, in the case of NagE, intracellular GlcNAc6P. The PTS transporters are composed of three (or occasionally four) domains [4]. In *E. coli* the *nagE* gene encodes all three domains of the GlcNAc specific PTS transporter (EIICBA^Nag^). The *nagE* gene forms part of a divergent operon system with four other genes, *nagE-nagBACD* (Figure 1A): *nagA* encodes GlcNAc6P deacetylase and *nagB* encodes GlcN6P deaminase, which together convert GlcNAc6P to fructose-6P, which enters the glycolytic pathway (Figure 1C) [5], [6]. NagC encodes the repressor of the operon and the function of NagD is unknown [7]. In *E. coli* glucosamine (GlcN) is transported by the generic hexose PTS encoded by *manXYZ* but its metabolism requires the *nagB* gene [4].

Of the two *nagE* homologues in *B. subtilis* identified by Reizer *et a*l [3], one, *yflF,* encodes a protein with just EIICB domains (50% identity with *nagE*_Ec_ in the longest aligned region and the other, *ybfS*, encodes a three-domain protein (EIICBA) (42% identity with *nagE*_Ec_). All % identities are given in Supporting Information, Table S1. The *ybfS* gene was located adjacent to a homologue of *nagB*_Ec_, *(ybfT*) and Reizer *et a*l [3] proposed that *ybfST,* constituted a module for uptake and use of GlcN. They showed that a *ybfS* mutant was impaired for growth on GlcN and called these two genes *gamA* and *gamP* for the glucosamine deaminase and transporter. An alternative nomenclature for *gamA* is *nagBB*. In addition Reizer *et al* [3] also showed that, contrary to *E. coli* [5], *B. subtilis* grows more rapidly on GlcN than GlcNAc and that a *ptsH* mutation inhibited growth on both sugars, confirming that they are transported by the PTS.

A second copy of a gene homologous to *nagB*_Ec_ is located adjacent to the *nagA* gene of *B. subtilis* (Figure 1B). The *gamA* and *nagB* encoded proteins of *B. subtilis* are 51% identical with each other and 37% and 40% identical to the *E. coli nagB* gene product (Table S1). Reizer et al [3] proposed that *nagAB*, together with the two domain transporter *yflS,* which they called *nagP,* were required for the metabolism of GlcNAc6P. (Confusingly, some databases refer to both the two-domain, *yflF*, and three-domain, *ybfS*, *nagE* homologues as *nagP*. To facilitate distinguishing the duplicated genes we have used the original *nagAB, nagP* and *gamAP* designations of Reizer et al [3], Figure 1). The third gene of the *nagAB* operon, *yvoA*, encodes a GntR family transcription factor. YvoA is homologous to the DasR protein, a pleiotropic regulator of use of GlcNAc and secondary metabolism in *Streptomyces coelicolor* [8]. Bertram et al [9] showed, by transcriptome analysis, that *nagA* and *nagP* expression was increased in a *yvoA* mutant, which they proposed to call *nagR*.

Amino sugar metabolism and its regulation have been well studied in *E. coli* [5], [10]–[12] and the structures and molecular mechanisms of the enzymes degrading them have been extensively analysed over the years: NagB, GlcN6P deaminase [13]–[16] and NagA, GlcNAc6P deacetylase [17]–[19]. Although the deaminase and deacetylase enzymes were partially purified from *B. subtilis* in the 1960′s [20]–[22], the enzymes were not further studied until recently. Vincent *et al* purified and solved the crystallographic structures of NagB_Bs_ and NagA_Bs_ [23], [24]. The enzymes were found to be similar to their *E. coli* homologues except that the NagB_Bs_ protein was found to be monomeric and non-allosteric whereas in *E. coli,* NagB_Ec_ is a homohexamer and allosteric [13].

In this work we have developed a novel system for targeted gene deletions in *B. subtilis* to systematically study the effects of inactivation of the *nag* and *gam* genes on growth on amino sugars. We show that, as predicted by Reizer *et al* [3], the *gamAP* operon is the major locus required for use of GlcN. However, although the *nagA* gene is essential for growth on GlcNAc, the majority of the GlcN6P deaminase activity during growth on GlcNAc is provided by GamA and not by NagB. We also show that NagP is the only transporter for GlcNAc, while GlcN is transported by GamP and also by PtsG, the major glucose transporter. We confirm the results of Bertram et al [9] that *yvoA*(*nagR*) regulates expression of the *nagAB* and *nagP* operons and show that *ybgA,* encoding another member of the GntR family of transcription regulators, and which is expressed divergently from *gamAP* (Figure 1B), represses expression of the adjacent *gamAP* operon.

## Materials and Methods

### Bacteriological Methods

Our wild type *B. subtilis* is SSB1002, a prototrophic (Trp^+^) version of 168. *B. subtilis* was grown in rich medium, LB, or in defined minimal MOPS medium [25] to which we added (NH_4_)_2_SO_4_ (20 mM) and ferric ammonium citrate (CAF) 11 µg/ml [26] and the other trace elements as recommended [27]. Carbon sources were added at 25 mM for glucose (Glc), GlcN and GlcNAc or at 0.8% K glutamate plus 0.6% Na succinate (KGS medium). Cas amino acids were at 0.5% where indicated. For growth experiments bacteria were grown overnight in minimal KGS medium, then diluted into fresh medium with Glc, GlcN or GlcNAc to an A_650_ of 0.01 at 37°C. Growth was followed by reading optical density (A_650_) and the growth rates were calculated from the log/linear regression of A_650_ versus time. *E. coli* strain IBPC118c (JM101 Δ*hsdR*) was used for transformations. Antibiotics were used at the following concentrations: *E. coli* ampicillin, 100 µg/ml in plates, 250 µg/ml in liquid except for pXE1-derived plasmids when 50 µg/ml was used for liquid and plates; spectinomycin, 50 µg/ml for *E. coli* and 100 µg/ml in *B. subtilis*; kanamycin, 25 µg/ml for *E. coli* and 5 µg/ml for *B. subtilis*; choramphenicol 5 µg/ml in *B. subtilis*; MLS, erythromycin 0.5 µg/ml and lincomycin 12.5 µg/ml in *B. subtilis*. Transformations were carried out by standard CaCl_2_ procedure for *E. coli* and by using the natural competence of *B. subtilis*. *B. subtilis* strains used or constructed in this work are listed in Table 1.

**Table 1:** *B. subtilis* strains used or constructed in this work.

Strain	Genotype	origin
SSB1002	WT *trp* ^+^ (equivalent to 1A2 from BCSC)	Lab stock H. Putzer
IG5c	Δ*gamP*	This work
IG7c	Δ*nagA*	This work
IG11c	Δ*nagP*	This work
IG17c	Δ*gamP* Δ*nagP*	This work
IG19c	Δ*gamAP*	This work
IG28c	Δ*yvoA(nagR)*	This work
IG30c	Δ*ybgA*	This work
IG50	*ptsI::kan*	This work
IG64c	Δ*ptsG*	This work
IG66c	Δ*gamP* Δ*ptsG*	This work
IG68c	Δ*gamP* Δ*nagP* Δ*ptsG*	This work
IG70c	Δ*yvoA(nagR)* Δ*ybgA*	This work
IG134c	Δ*gamA*	This work
IG136c	Δ*nagB*	This work
IG138c	Δ*gamA* Δ*nagB*	This work
IG249	Δ*ypqE*::FRTspec	This work
IG251	Δ*gamP* Δ*ptsG* Δ*ypqE*::FRTspec	This work
IG312	Δ*yvoA(nagR)* Δ*gamP*	This work
IG314	Δ*ybgA* Δ*nagP*	This work
BSB168*ptsI*::kan	*ptsI*::kan	J. Deutscher

### Construction of a Plasmid Carrying an Excisable Spectinomycin Cassette

**Figure 2:**
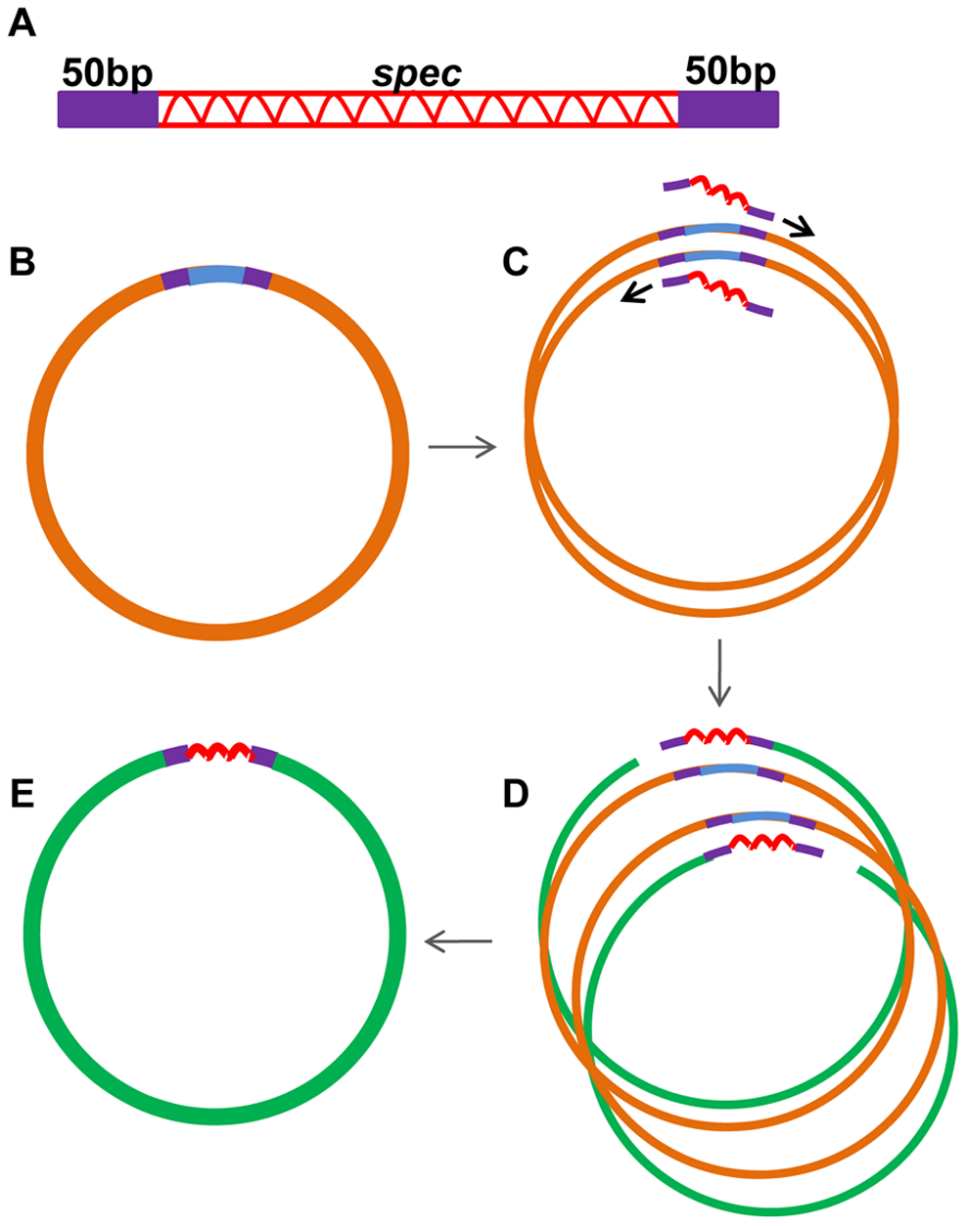
Construction of gene replacement mutations by Overlap Extension PCR (OEPCR). A. DNA fragment carrying the *spec* cassette (shown as red wavy line) with 50 bp targeting sequences at either end is denatured and annealed with a plasmid (B) carrying the gene to be replaced. Extension of the two strands of the fragment on both strands of the plasmid (C) produces full size single-stranded plasmid DNA with a nick at either end of the targeting sequence (D), which can anneal together. This process is repeated for 30 cycles of the OEPCR. Transformation into *E. coli* selecting for *spec* resistance repairs the nicks and produces full size plasmid DNA where the targeted gene has been replaced by the *spec* cassette (E).

The ps4-ps1 PCR fragment of pKD13, carrying a kanamycin resistance cassette (*kan*) surrounded by FRT (Flp recombination target) sites recognised by the Flp recombinase from the 2 micron plasmid [28], was amplified and inserted into pBR322 digested with EcoRV. This gave plasmid, pBR/ps4-1FRTkan, which is replicable in a wild-type (*pir* ^+^) *E. coli* strain. The *kan* cassette (non-functional in *B. subtilis*) was replaced by the spectinomycin resistance cassette (*spec*) from pDG1727 [29], which is expressed in both *E. coli* and *B. subtilis*. The whole spectinomycin cassette, with promoter and flanking sequences (1.2 kb), was amplified with the oligonucleotides 5′kanspec and 3′kanspec (Table S2), where the 25 nt at the 3′ end of the oligonucleotides are homologous to the ends of the *spec* cassette and the 50 nt at the 5′ end are homologous to the 5′ and 3′ extremities of the *kan* cassette of pKD13. These 50 bp extensions were used as targeting sequences to the ends of the *kan* cassette of pBR/ps4-1FRTkan to replace the *kan* cassette with the *spec* cassette by overlap extension PCR cloning (OEPCR) [30], as described in Figure 2. The purified *spec* fragment was used as a mega-primer and added in 50–250 fold molar excess to the pBR/ps4-1FRTkan plasmid (25 ng), with 0.2 mM NTPs in the supplied HF buffer with the enzyme Phusion (1 unit) (Fermentas) in a final volume of 10 µL. Polymerisation of the mega-primer DNA fragment directs the synthesis of the two strands of the plasmid. This corresponds to an arithmetic and not logarithmic amplification. The PCR program used was 98°C for 2 min; then 30 cycles of 98°C for 30 sec, 43°C for 30 sec, 68°C for 1.5 min/kb plasmid [30]. The PCR mixture was used to transform IBPC118c selecting for ampicillin and spectinomycin resistance and screening for kanamycin sensitivity. The candidate colonies were screened by mini-PCR using an oligonucleotide hybridizing to the plasmid vector and an oligonucleotide specific to the *spec* insert (ps1 or ps4) to identify correct clones. The resulting plasmid pBR/ps4-1FRTspec was purified and used as a template for amplifying the *spec* gene surrounded by FRT sites. The number of transformants with 2 µl of OEPCR mix varied from about 10 to several hundreds depending upon the plasmid and fragment to replace.

### Construction of Spectinomycin Resistant Replacements of the *nag* and *gam* Genes of *B. subtilis*

DNA fragments of *B. subtilis* chromosome carrying the *ybgA-gamAP, hprK-nagAByvoA, nagP*, *ptsG* and *ypqE* loci with about 500 bp flanking sequences were amplified by PCR using Phusion (Fermentas) and the pairs of oligonucleotides given in Table S2. The purified DNA fragments were inserted into pJRD184 [31] to give pJRD/ybgAgamAP and pJRD/hprKnagAByvoA or into pXEI [32] to give pXE/nagP, and pXE/ptsG, normally using the enzymes whose restriction sites were incorporated into the oligonucleotides (Table S2). The *nagP* gene was inserted, as a StuI to NaeI fragment from the 5′NagPNco1 to 3′NagPBamHI PCR fragment, into pXE1 digested with EcoRI and made blunt with T4 polymerase. Shorter version of the *nagAByvoA* insert was made by cloning the BsNag4-BsNag8 fragment of pJRD/hprKnagAByvoA into pJRD184 digested with EcoRV. The entire insert of each plasmid was verified by sequencing using suitably spaced internal primers (MWG Eurofins).

The *spec* cassette surrounded by FRT sites, was amplified from pBR/ps4-ps1FRTspec with oligonucleotides carrying at their 5′ ends 50 bp targeting sequences for each of the *gam*, *nag* and *ptsG* genes and at their 3′ ends the ps4 or ps1 sequences using Advantage HD enzyme (Clontech). The 5′ targeting oligonucleotide sequence corresponded to 50 bp upstream of and including the initiation codon of the gene to be replaced and the 3′ targeting sequence included the last 6 amino acids of the protein and DNA further downstream of the STOP codon. To ensure that the "scar" sequence left after curing the *spec* cassette, is in phase with the original start and stop codons of the replaced gene an A was added to the ps4 sequence and AT to the ps1 sequence in these targeting oligonucleotides. The pairs of oligonucleotides used are listed in Table S2. The *spec* cassette fragments with 50 bp targeting sequences at their 5′ and 3′ ends were used as the mega-primers for OEPCR (as described above, Figure 2), using the plasmids carrying the cloned *gam/nag/ptsG* regions as templates to replace the individual genes with the *spec* cassette. The OEPCR mixture was used to transform IBPC118c selecting for Amp and Spec resistance. Candidate transformants were screened by mini PCR as above and plasmid DNA purified. Plasmids (non-replicative in *B. subtilis)* carrying the correct *spec* replacement were linearized and used to transform competent *B. subtilis* selecting for Spec resistance. Double recombination replaced the targeted gene at its chromosomal locus with the *spec* cassette and the replacement was verified by mini-PCR on purified transformed colonies using oligonucleotides (ps1 or ps4) specific to the cassette and hybridizing upstream or downstream of the replaced gene.

### Construction of a Plasmid Expressing the Flp Recombinase in *B. subtilis* and Curing of the *spec* Cassettes

The 1679 bp EcoRI to BamHI fragment carrying the pSpac-lacI cassette from pDG148 [33] was inserted into pYH224 [34] to give pYH/pSpac. pYH224 carries a thermosensitive replicon and confers MLS resistance. The *flp* gene encoding the Flp recombinase of the 2 micron plasmid was amplified from pCP20 [28], with the oligonucleotides 5′FlpAflII and 3′FlpSbfI and inserted into pYH/pSpac, digested with AflII and SbfI, downstream of the pSpac promoter, to give pYH/pSpac-FLP. It was transformed into the *B. subtilis* strains carrying the *spec* replacements selecting for MLS resistance at 30°C. The transformants were purified once at 42°C on LB plates with 0.5 mM IPTG to simultaneously activate expression of Flp and permit loss of the pYH224 derived plasmid. Resulting bacteria were purified on LB plates at 37°C and tested for Spec and MLS sensitivity. Loss of the cassette was verified by PCR and the whole of the replaced region verified by sequencing a PCR fragment covering the deletion. An alternative Flp delivery plasmid based upon a xylose inducible promoter (pYH/pXyl-FLP) was also constructed.

### mRNA Preparation, Northern Blots and Primer Extension Assays

Bacteria were grown overnight in MOPS KGS medium with 0.5% cas amino acids and then diluted into fresh medium with either Glc, GlcN GlcNAc or KGS as carbon source. Bacteria (25 ml) were harvested at an OD650 of about 0.8 and frozen at −20°C. RNA was extracted by the glass beads and cold phenol method [35]. RNAs were separated on 0.8% agarose gels in 1× TBE and transferred overnight to HyBond N+ membranes by downward capillary transfer [36] in 5× SSC with 10 mM NaOH. The probes used were oligonucleotides of approximately 30–40 nt (Table S2) labelled at their 5′ end by polynucleotide kinase and γ[^32^P]ATP. Hybridization was carried out overnight using UltraHyb-oligo medium (Ambion) at 42°C. After washing, hybridization patterns were analysed by phosphoimagery and transcripts quantified with the ImageQuant program. The blots were rehybridized with a probe for 5S RNA as a loading control. The 5′ ends of the *gamA, nagA* and *nagP* genes were mapped by primer extension basically as described [37], using the oligonucleotides listed in Table S2.

## Results

### An Excisable Spectinomycin Cassette which Functions in *B. subtilis*

To facilitate the construction of multiple deletions within the *nag* and *gam* genes on the *B. subtilis* chromosome, we constructed a system of excisable antibiotic resistance cassettes in *B. subtilis* analogous to, and based upon, the Datsenko and Wanner system in *E. coli* [28]. FRT (Flp recombination target) sites, which are recognised by the Flp recombinase enzyme, were placed around a spectinomycin resistance gene (*spec*). This cassette was used to replace the various *B. subtilis gam* and *nag* genes carried on plasmids in *E. coli* by an overlap extension PCR (OEPCR) method [30] (Figure 2). A DNA fragment consisting of the FRT-*spec* cassette with 50 bp extensions targeting the translational initiation and termination regions of the gene to be replaced, was synthesized by PCR using oligonucleotides with the 50 nt targeting extensions at their 5′ ends. These fragments were used as the "mega primer" for OEPCR with a plasmid carrying the wild-type *gam* or *nag* genes as template. The product from OEPCR was used directly to transform *E. coli* selecting for ampicillin and spectinomycin resistant plasmids, where the FRT-*spec* cassette has replaced the *gam* or *nag* gene. Plasmid DNA carrying the *spec* replacements was transformed into *B. subtilis* (wt or carrying previously introduced mutations at distal sites) to replace the corresponding chromosomal gene with the *spec* resistance gene by homologous double recombination. In general the whole of the gene was replaced between the translation initiation codon and the last 6 amino acids of the protein.

The *spec* cassette was subsequently removed by transformation with a *B. subtilis* plasmid (pYH/pSpacFLP) with a thermosensitive replicon and expressing the recombinase (Flp) gene from the yeast 2 micron plasmid under control of the pSpac promoter. Purification of the transformants at 42°C in the presence of IPTG allowed simultaneous loss of the antibiotic cassette and the Flp-expressing plasmid. After excision a scar sequence is left, which forms part of a short ORF (34 aa), which starts at the ATG codon of the deleted gene and terminates at its stop codon, if the initial homing oligonucleotide primers are chosen judiciously. The presence of this small ORF should minimize any effects of polarity on transcription of any downstream genes.

### Effect of Deletions in the *gam* and *nag* Genes on Growth on Amino Sugars

**Figure 3:**
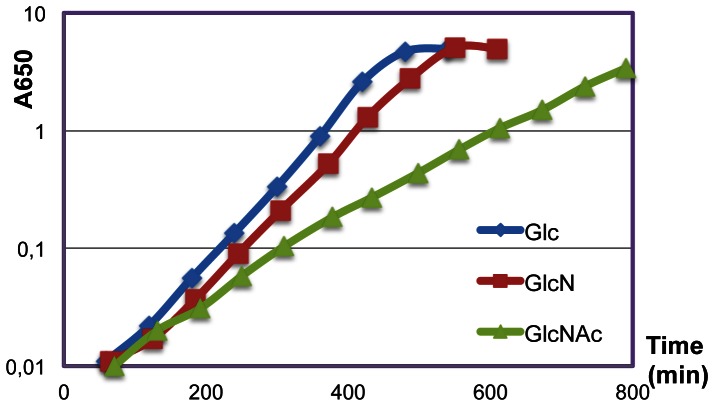
Growth of *B.* subtilis on GlcN and GlcNAc. Wild-type *B. subtilis* was grown overnight in KGS medium and then diluted into minimal medium with glucose, GlcN or GlcNAc as carbon source at an initial A650 = 0.01 and growth monitored by following OD.

**Figure 4:**
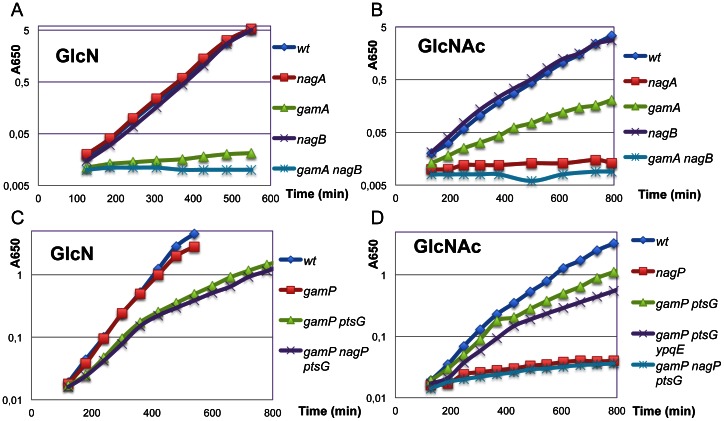
Effect of mutations on growth on GlcN and GlcNAc. Mutations in genes for the catabolic enzymes (A, B) and transporters (C, D) of the *nag* and *gam* operons on growth on GlcN (A, C) and on GlcNAc (B, D). Bacteria carrying the mutations indicated were grown overnight in KGS medium and diluted into minimal medium with GlcN or GlcNAc as carbon source at A650 = 0.01 and growth monitored by following OD.

Each of the *nag* and *gam* genes was deleted individually by this method and combinations of deletions constructed by introducing a second and third mutation into the cured strains. Growth rates of the different deleted strains were measured during growth in minimal medium with glucose, GlcN and GlcNAc. All strains grew identically on glucose (Doubling time (DT) = 44±2 min). Wild-type *B. subtilis* grew almost as rapidly on GlcN as on glucose (DT about 48 min) while growth on GlcNAc was significantly slower (DT about 93 min) (Figure 3). (All DT are given in Table S3). As expected deletion of the *nagA* gene prevented all growth on GlcNAc (no growth after 12 h) but had no effect on growth on GlcN (Figure 4A, B). Deletion of the *nagB* gene, located downstream of *nagA* (Figure 1B) had essentially no effect on growth on either GlcN or GlcNAc. On the other hand, deletion of *gamA,* encoding the second copy of GlcN6P deaminase, prevented nearly all growth on GlcN (DT about 8h) and severely reduced growth on GlcNAc (DT about 3h). The strain with the double deletion of *gamA* and *nagB* did not grow on either sugar (Figure 4A, B). Thus the *gamA* gene is not only required for growth on GlcN but also must supply the majority of GlcN6P deaminase activity during growth on GlcNAc.

**Figure 5:**
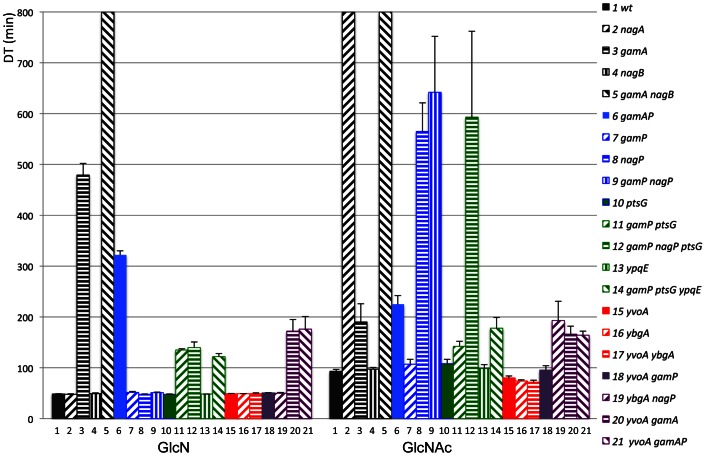
Effect of mutations in enzymes, transporters and regulatory genes on growth on GlcN and GlcNAc. Bacteria were grown in minimal medium with GlcN or GlcNAc as in Figures 3 and 4. Doubling times were calculated by log/linear regression of the OD compared to time generally in the range A_650_ of 0.1–1.5 OD. Values are means with standard errors for 2 to 4 cultures. The *nagA* strain (column 2) on GlcNAc and the *gamA nagB* strains (column 5) on GlcNAc and GlcN did not grow during the 12 h period of the experiment and are shown as >800 min. Strains with mutations in genes for enzymes are shaded in black, in transporters in blue or green, in regulators in red, and in combinations of regulators with enzymes or transporters in violet. The *yvoA* gene has been called *nagR* [9]. The numerical values of the doubling times are given in Table S3.

The genes for the two putative amino sugar PTS transporters, *gamP* and *nagP* were also deleted. Surprisingly deletion of *gamP* had only a minimal effect on growth on either amino sugar, while deletion of *nagP* almost completely prevented growth on GlcNAc (DT about 9 h) but had no effect on growth on GlcN (Figure 4C and D). The double deletion *gamP nagP* still grew normally on GlcN implying that another transporter was involved in transport of GlcN (Figure 5 columns 7–9). This transporter should also be a PTS type since a *ptsI* mutation (*ptsI::kan* from J. Deutscher) prevented all growth on both sugars. Reizer *et al* [3] had previously observed that a *ptsH* mutation inhibited growth on both sugars. The closest homologue to *gamP* in *B. subtilis* is the glucose transporter *ptsG* (EIICBA^Glc^) (50% identity, Table S1) [3]. Deletion of this gene reduced the growth rate on glucose (DT = 75 min) but alone had no significant effect on growth on GlcN. However the double mutation *gamP ptsG* was distinctly slower on GlcN (DT 135 min). The triple mutant, *gamP, nagP, ptsG* grew similarly to or only slightly more slowly than *gamP, ptsG* on GlcN (Figure 4C), showing that *nagP* can contribute only marginally to growth on GlcN (Figure 5 columns 10–12). However the fact that the triple mutant *gamP nagP ptsG* still grew with a DT of about 140 min implies that some other PTS transporter can transport GlcN. Overall these data show that the *gamAP* operon and, in particular the *gamA* gene, is responsible for the good growth of *B. subtilis* on GlcN.

On the other hand NagP appears to be the only transporter for GlcNAc since it essentially eliminates growth on GlcNAc and the inclusion of *nagP* and *ptsG* mutations does not affect the residual growth (Figure 4D). However, in a *nagP* ^+^ strain we noted that *gamP* and *ptsG* mutations did decrease growth on GlcNAc (DT = 140 min) compared to the wild-type. The *nagP* gene encodes an Enzyme II with just the EIICB domains and so NagP requires an EIIA domain to transfer the phosphate from HPr to its EIIB domain. Both *ptsG* and *gamP* encode EIICBA proteins and could supply the EIIA function, which might explain their slight effect on the growth rate in a *nagP* ^+^ strain. However the majority of the IIA function must be supplied by another EIIA. The *B. subtilis* chromosome carries a gene for an isolated EIIA protein, *ypqE* [3], homologous to both *gamP* and *ptsG* EIIA domains (50% and 46% identity respectively). The *ypqE* gene was also deleted and the effect of its deletion on growth on GlcNAc, both alone and in combination with *gamP ptsG* mutations, tested. Alone it had no significant effect on growth on GlcNAc, but in combination with *gamP ptsG,* growth was further reduced (DT 180 min, Figure 4D, Figure 5 columns 13,14). This is still much faster than growth of the *nagP* mutant, so that although NagP is essential for growth on GlcNAc, it can receive a phosphate from several EIIA domains. YpqE, PtsG and GamP can all supply NagP EIIB domain with phosphate but another unknown protein is probably supplying the major EIIA function for NagP. (See Figure 5 and Table S3 for all doubling times.).

### YvoA(NagR) and YbgA Regulate Expression of the *nag* and *gam* Genes Respectively

Two genes for GntR family transcription factors are associated with the *gamAP* and *nagAB* operons (Figure 1B). The *yvoA* gene has been shown to control expression of *nagA* and *nagP* and was consequently called *nagR* [9]. We created a deletion in *ybgA* and compared its effect to that of a *yvoA(nagR)* deletion on growth on GlcN and GlcNAc and on expression of the *nag* and *gam* genes with Northern blots.

**Figure 6:**
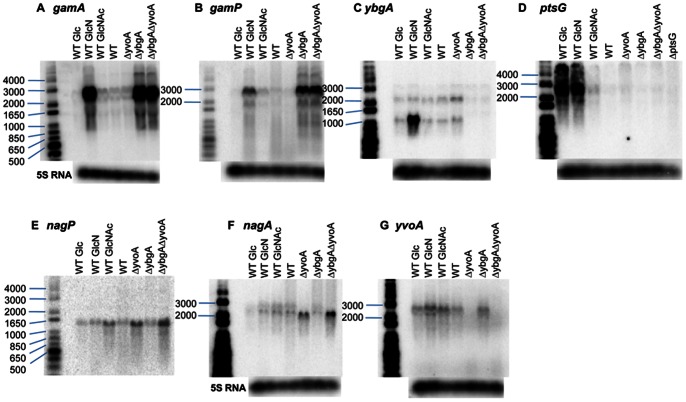
Transcription of the *nag* and *gam* genes of *B.* subtilis. RNAs (10 µg) prepared from wild-type *B. subtilis* grown on the sugars indicated (Glc, GlcN, GlcNAc) or wild-type and mutant bacteria grown in KGS medium, were separated on a 0.8% agarose gel run in TBE buffer. After transfer to Hybond-N+ membranes, they were hybridized with probes for the genes indicated (Table S2) and analysed by phosphoimagery. The *yvoA* gene has been renamed *nagR* [9]. The marker is 1 kb ladder labelled with [γ^32^P]-ATP and polynucleotide kinase. After stripping the hybridized probe, the membranes were rehybridized with a probe for 5S RNA as a loading control.

A transcript compatible with the size of the *gamAP* operon (2.7 kb) was strongly expressed during growth on GlcN or in the presence of the *ybgA* mutation (Figure 6A). The increase in expression produced by the *ybgA* mutation was at least 50 fold compared to wild-type bacteria grown in KGS medium. A transcript of the same size was detected using a *gamP* probe (Figure 6B). These observations suggest that *ybgA* encodes the repressor of the *gamAP* operon and, moreover, are consistent with the idea that the operon is dedicated to the use of GlcN [3]. Expression of the divergently expressed *ybgA* gene was also increased by growth on GlcN (about 10 fold) (Figure 6C). Two transcripts were detected with the *ybgA* probe; one, of about 1000 nt, could correspond to either the *ybgA* gene alone (750 bp) or (more likely) to the *ybgAB* operon (1060 bp) including a small ORF downstream of *ybgA* (Figure 1B) The longer transcript (>2000 nt) might correspond to read through into the downstream *ilvE* gene. It could also correspond to an antisense transcript initiated within the *gamAP* genes. High resolution transcriptome mapping using tiling arrays, located the 5′ end of an antisense RNA (annotated as U167.M7) within the beginning of *gamP* and it would produce a transcript of about 2000 nt if it terminated after *ybgAB* [38]. The longer *ybgA* transcript was not detected in the *ybgA* mutant, showing that it is not due to cross-reaction with another transcript. Interestingly only the short *ybgA* specific transcript is induced by growth on GlcN, which is more compatible with the longer transcript corresponding to expression from the upstream antisense promoter within *gamP*.

Using a probe for *nagP,* a transcript corresponding to the size of the *nagP* gene (1.4 kb) was detected (Figure 6E). Its expression increased about 3- to 4-fold during growth on GlcNAc or in the presence of the *yvoA*(*nagR*) mutation. A transcript comparable in size to the *nagAB* operon (1.95 kb), was detected by the *nagA* probe (Figure 6F). A similarly sized transcript, but slightly stronger, was detected in strains deleted for *yvoA* (grown in KGS medium) and in this strain must correspond to just the *nagAB* genes since the downstream *yvoA* gene is deleted except for the scar (84 bp) left after excision of the FRT cassette. A *yvoA* probe (Figure 6G) detected a weak transcript of about 3000 nt, which should correspond to the *nagAB yvoA* operon (2.7 kb). The transcript appears as a doublet but this could be an artefact because it is a low abundance transcript running very near to the very abundant 23S rRNA (2904 nt). The faint, somewhat diffuse, signal above the approximate 2000 nt transcript detected with the *nagA* probe, could correspond to the full length *nagAB yvoA* transcript (Figure 6F). Since the major transcript detected with the *nagA* probe in *yvoA* ^+^ strains is the same size as the *nagAB* transcript in Δ*yvoA* strains, the tricistronic *nagAB yvoA* mRNA is probably processed to a shorter *nagAB* mRNA and the downstream *yvoA* part is rapidly degraded. No shorter transcripts corresponding to the size of the *yvoA* gene (750 bp) were detected, strongly suggesting that *yvoA* is expressed only as part of the *nagAB yvoA* operon. These data are consistent with YvoA(NagR) being the specific regulator of the *nagAB* and *nagP* genes as proposed by Titgemeyer and colleagues [9], [39].

The 5′ ends of the *nag* and *gam* transcripts were mapped by primer extension and their positions could be correlated with possible sigma A dependent promoter sequences (Table 2). The intensity of the primer extension signals from RNA prepared from bacteria growing on Glc, GlcN or GlcNAc (Figure S1) mirrored the pattern of expression of genes monitored by Northern blot.

**Table 2:** Mapped 5′ ends of transcripts.

gene	−35 - spacer length −10^a^	−10 to +1^b^	+1 to ORF^c^
*gamA*	TTGACA -18- TAAATT	8	63
*ybgA*	TTTCAT -17- TATTAT	7	41
*nagA*	TAGATC -17- TAAAAT	7	29
*hprK*	TTTAGG -17- TAAAAT	7	44
*nagP*	TTGGTA -17 - TATATG	7 d	30 d

a sequence of proposed −35 and −10 consensus sequences and distance (bp) between them.

b Distance (bp) between the last base of the −10 hexamer and the mapped transcription start site (+1).

c Distance (bp) between +1 and initiation codon of gene.

d Estimation from pBR322 MspI markers.

The 5′ ends were mapped by primer extension on mRNA prepared from bacteria grown in glucose, GlcN or GlcNAc using the oligonucleotides indicated in Table S2. The same oligonucleotides were used to generate sequencing reactions, which were run in parallel. (The gels are shown in Figure S1).

### *ptsG* and *hprK* are not Regulated by *yvoA(nagR)* or *ybgA*

As *ptsG* is capable of efficient transport of GlcN, we examined its mRNA level during growth on GlcN. A probe for *ptsG* detected two transcripts, one of about 2500 nt, which could correspond to just the *ptsG* transcript, and another of greater than 4000 nt, which presumably corresponds to the *ptsGHI* transcript (Figure 6D) [40]. These transcripts were strongly induced by growth on glucose and on GlcN but not by the presence of the *ybgA* or *yvoA(nagR)* mutation. Glucose increases *ptsGHI* expression by activating the GlcT transcription anti-terminator. Normally growth on glucose dephosphorylates PtsG, which in turn activates the GlcT anti-terminator via its dephosphorylation [40]. The increase in expression by glucosamine could be due to cross regulation of GlcT, during growth on another PTS sugar. Growth on GlcN dephosphorylates GamP, which could derepress the *ptsG* gene either by directly activating (dephosphorylating) GlcT or indirectly by provoking the dephosphorylation of PtsG via HPr [41].

The *hprK* gene, encoding the kinase responsible for the regulatory phosphorylation of HPr involved in carbon catabolite repression [41], is expressed divergently from the *nagAB yvoA* operon (Figure 1B) and previous work suggested it was expressed constitutively [42]. We localized the 5′ end of *hprK* by primer extension (Table 2 and Figure S1). The 5′ ends of the *nagA* and *hprK* transcripts are separated by only 110 bp. The *hprK* transcript was detected in all three media, glucose, glucosamine and GlcNAc and was not affected by the presence of *ybgA* or *yvoA* mutations, showing that *hprK* is not part of the amino sugar regulon in *B. subtilis*.

### Mutations in both *yvoA* (*nagR*) and *ybgA* Improve the Growth Rate on GlcNAc

The presence of a *ybgA,* a *yvoA* or the double *ybgA yvoA* mutation produced significant increases in the growth rates on GlcNAc with DT of about 70–80 min (Figure 5 columns 15–17, Table S3). The increase in growth rate produced by *yvoA* can be understood as corresponding to an increase in *nagP* and/or *nagAB* expression [9] (Figure 6 E, F) and hence uptake and metabolism of GlcNAc. The fact that the *yvoA* mutant grows better on GlcNAc than the wild-type strain indicates that GlcNAc does not result in full induction of the *nagAB* and *nagP* operons, as observed by Bertram et al [9]. The effect of the *ybgA* mutation could be due to increased expression of *gamA*, since GamA is the major source of deaminase activity during growth on GlcNAc (Figures 4B, 5) and growth on GlcNAc only produces a slight increase in *gamAP* expression (Figure 6A). The strain with the double mutation, *ybgA yvoA,* was only very slightly faster than either of the single mutations (Figure 5 columns 15–17).

It can also be noted that, although GamP does not appear to contribute to growth of the wild-type strain on GlcNAc, since alone the *gamP* mutation has no effect and the *nagP* mutation essentially stops all growth on GlcNAc (Figure 4D), the *ybgA nagP* strain, was capable of appreciable growth on GlcNAc (DT about 190 min, Figure 5 columns 19, Table S3). In this strain expression of *gamAP* is strongly induced by the *ybgA* mutation, implying that the GamP transporter, when expressed, can transport GlcNAc. Similarly combinations of *yvoA* and *gamA* or *gamAP* mutations allow better growth on GlcN (DT 170 min) compared to the *gamA* or *gamAP* mutations alone (DTs of 450 and 320 min) (Figure 5 compare columns 3, 6 with 20, 21). Thus the NagB encoded deaminase does allow growth on GlcN but only when its expression is derepressed by a *yvoA* mutation. Growth on GlcN does not relieve YvoA (NagR) repression. Similarly growth on GlcNAc had little effect on YbgA repression of *gamAP.* The *gamAP* mRNA did increase slightly during growth on GlcNAc (Figure 6A,B), which can be explained by the production of GlcN6P from the catabolism of GlcNAc (Figure 1C).

### Growth of other *Bacillus* Species on GlcN and GlcNAc

**Figure 7:**
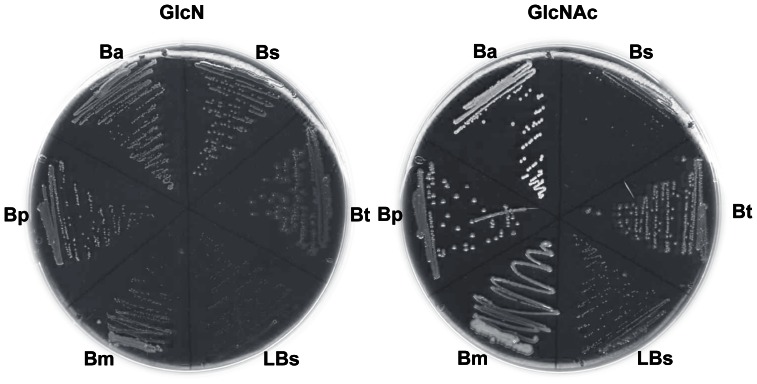
Growth of other *Bacillaceae* on GlcN and GlcNAc. Bacteria were streaked from LB plates to M9 plates with thiamine (10 µg/ml), biotin (1 µg/ml) and 10 mM GlcN or 10 mM GlcNAc. B*s B. subtilis SSB1002;* Bm *B. megaterium* QMB1551, Bp *B. pumilus* 8A1, Bt *B. thuringiensis* 407cry- [54], Ba *B. amyloliquefaciens*, FZB42 [55], LBs *Lysinibacillus sphaericus*. The bacteria were grown for 48 h at 37°C.

To investigate if the preference for growth on GlcN was characteristic of *Bacillus* species in general, we tested the capacity of a representative selection of strains to grow on GlcN and GlcNAc (Figure 7). We have not tried to optimize growth conditions for these other strains and their relative growth rates varied considerably. All the five species tested grew as well or better on GlcNAc than on GlcN on solid media. *B. subtilis* grows relatively well on both solid media, although the colonies are larger on GlcN than GlcNAc, as expected from the difference in DT. *B.* *megaterium* in particular grew very well on GlcNAc plates and growth in liquid was as rapid on GlcNAc as on Glc (DT about 40 min) while it grew much more slowly on GlcN (DT about 200 min). *B. pumilus* grew on both GlcN and GlcNAc in liquid media with DT of about 120 and 90 min respectively, a little slower than on Glc (DT 70 min). *B. amyloliquefaciens* on the other hand grew with a similar DT to *B. pumilus* on GlcNAc in liquid media but failed to grow on GlcN (although growth was detectable on the GlcN agar plates). *B. thuringiensis* grew slightly better on solid GlcNAc medium than GlcN but failed to grow in liquid on either amino sugar or glucose. *Lysinibacillus sphaericus* hardly grew after 2 days on plates on either sugar but the residual growth observed was stronger on GlcNAc than GlcN. Although usually thought to not use hexoses or pentoses as carbon sources, a GlcNAc PTS operon was shown to be functional in *B. sphaericus* 2362 [43].

## Discussion

### Growth of *B. subtilis* on GlcN

The deletion analysis of *nagAB, nagP* and *gamAP* genes has confirmed the gene function predictions based on sequence homology of Reizer *et al* [3] but with certain modifications. The efficient use of GlcN by *B. subtilis*, producing a doubling time comparable to that during growth on glucose, can be correlated with the high expression of the *gamAP* operon and in particular *gamA*. Although Reizer *et al* [3] found that their insertion mutant in *gamP* was impaired in growth on GlcN, we found no phenotype for our *gamP* deletion and showed that *ptsG* is capable of replacing *gamP.* Growth on GlcNAc requires *nagA* and *nagP* gene products but unexpectedly relies upon the *gamA* encoded deaminase rather than the *nagB* gene, cotranscribed with *nagA*. The relatively poor growth on GlcNAc can be ascribed to the low level of *nagA* and *nagP* transcript. Since the *yvoA(nagR)* and/or *ybgA* mutant strains grow more rapidly on GlcNAc than the wild-type, we can deduce that growth on GlcNAc does not lead to full derepression of the *nagAB* and *nagP* operons as observed previously [9]. However even the *ybgA yvoA* strain grows significantly more slowly (DT about 70 min) than wild-type *B. subtilis* on GlcN (48 min).Our analysis of deletion mutations in *yvoA(nagR)* and *ybgA* genes has confirmed that YvoA/NagR encodes a repressor for the *nagP* and *nagA* genes [9] and shown that YbgA is responsible for the transcriptional repression of the *gamAP* operon. Our results fully support those of Bertram *et al* [9] and Resch *et al* [39] who found consensus sequences for the YvoA(NagR) protein upstream of *nagA* and *nagP*, analogous to the *dre* sites identified for DasR, the regulator of GlcNAc use in *Streptomyces coelicolor* [8]. Their YvoA(NagR) operator consensus sequences overlap the −35 sequences of the promoters identified by 5′ end mapping of the *nagA* and *nagP* transcripts. Our results also demonstrate that *ybgA* encodes a repressor of the *gamAP* operon and DNA binding experiments show that YbgA is the direct repressor of the *gamAP* operon (unpublished data). There seems to be little cross regulation of the *nag* genes by growth on GlcN or of the *gamAP* operon by growth on GlcNAc.

The transcripts characterized in this work find their counterparts within the global *B. subtilis* tiling array transcriptome data of Nicolas *et al* [38], although none of the conditions analysed in that work are expected to have induced the expression of the *gam/nag* operons and so only basal expression was detected. Their analysis indicated that *gamAP* operon terminates before the downstream *gltP* gene while the *ybgA* transcript includes the small *ybgB* gene, which is in agreement with the size of the transcript detected by Northern blot (Figure 6C). The *ybgB* gene encodes a small, uncharacterized protein, which was previously shown to be transcribed from a SigY-dependent promoter [44]. The transcriptome analysis also detected RNA expression corresponding to the *nagAB yvoA* operon and *nagP* gene.

### The *gamAP* Operon is Unique in *B. subtilis* amongst all Firmicutes

Synteny analysis of the *nagA, nagB, nagP, ptsG, yvoE, ypqE* genes using the Absynte [45] and SyntTax [46] programs shows that these genes are present in essentially all the sequenced genomes of *Bacillaceae* (Figure S2 A,B,C,D,E,F). The exact organisation of the surrounding genes is not always conserved but the three genes forming the *nagAB yvoA* synton are frequently found in close proximity, not only in the *Bacillaceae* family [9] (Figure 1B, Figure S2 A, B, F) but also in the remaining *Firmicutes* and in several sequenced genomes belonging to the *Synergistetes*, *Actinobacteria* and *Proteobacteria* phyla (data not shown). They are however absent from the facultative anaerobe and thermophilic *Anoxybacillus flavithermus.*

In *B. subtilis nagAB* are expressed divergently from *hprK* but only in *B. pumilus* and *B. amyoliquefaciens* is this configuration retained (Figure 1B). In *B. megaterium* the *nagAB yvoA* genes are expressed divergently from a gene, *nagE*, encoding a three-domain (EIICBA) transporter, homologous to the *nagE* gene from *E. coli.* In all the other *Bacilli,* the gene with the highest homology to *nagE*_Ec_, *nagP*, encodes just the EIICB domains (Figure S2C). In *B. halodurans, nagAB yvoA* are expressed divergently from this IICB domain protein, *nagP,* and in *B. licheniformis nagP* is two genes downstream of *nagAB yvoA*. In all the other *Bacilli nagP* is present as an isolated gene, distant from *nagAB* and it has diverged in both location and sequence. In *B. thuringiensis* and *B. cereus* the *glcT ptsGHI* operon is found one gene downstream of *nagAB yvoA*. These organisms possess, in addition, a *nagP* homologue (Figure 1B). The *ptsG* genomic context (*glcT ptsG ptsH ptsI*) is well conserved (Figure S2D). The gene encoding the lone EIIA protein, *ypqE,* is also ubiquitous but in a very variable genomic context (Figure S2E).

On the other hand, searching for homologues to *gamA* or *gamP* shows this operon together with the divergently expressed *ybgA*, is only present in *B. subtilis* (Figure S2G, H, I). It is not found in any other sequenced firmicute or indeed any other bacterial or archaeal species. The closest homologues to GamA and GamP are NagB and PtsG in all other *Bacillaceae* (Figure S2G, H). The closest homologue to YbgA is YvoA in most but not all species (Figure S2I). *B. subtilis* has thus acquired a novel synton, *ybgA-gamAP*, that enables it to use glucosamine efficiently as a carbon source. In order to investigate whether this new operon originated from an endogenous duplication of the *nagAB yvoA* and *nagP* operons or by lateral gene transfer, we performed a detailed synteny analysis [46] of this region in the 64 sequenced species composing the *Bacillus* genus. The synteny centered on the *ybgA* gene indicated the presence of an additional gene for a small (91 amino acids) protein of unknown function, *ybgB,* closely linked to the operon in all the 9 fully sequenced *B. subtilis* genomes. The *ybgB ybgA-gamA* grouping is also found in *B. atropheus* but the *gamP* gene is almost completely missing in this organism, presumably due to a subsequent deletion event. Incomplete genome sequences of *B. vallismortis* and *B. mojavensis* genomes also have the *ybgB ybgA-gamAP* synton with high amino acid sequence identity to that of *B. subtilis,* which is consistent with their being close neighbours of *B. subtilis* [47]. The *ybgB* gene is also found in *B. amyloliquefaciens,* but in a different gene context, while the *ybgA-gamAP* genes are absent from this organism. There are no other close homologues of YbgB within sequenced genomes (Figure S2J). The *ybgBA gamAP* synton is thus a mixture of duplicated conserved genes (presumably the result of an ancient genome rearrangement) and a single copy gene *ybgB*, with very limited penetration in other genomes. The latter might possibly be an artifact that was produced at the time of the genome rearrangement, in which case it is likely to be non-functional and might have been selected against. Intriguingly, the *ybgB* gene was found to be the only other target promoter of the extracytoplasmic Sigma Y (itself of unknown function) [44]. The presence of a unique *ybgB* gene linked to the glucosamine utilization functions in *B. subtilis,* therefore argues against a recent gene duplication event and suggests that the entire *ybgB*-*ybgA-gamAP* synton was acquired by horizontal gene transfer from a yet unknown donor. The function of the small YbgB protein remains unknown. Its interruption had no obvious effect on growth on GlcN or GlcNAc.

The absence of the *ybgA-gamAP* genes can be correlated with the preferential growth of the other 5 *Bacillus* species tested for growth on GlcNAc (Figure 7). However it should be noted that most of those tested grew faster on glucose than GlcNAc except *B.* *megaterium* (DT about 40 min on both sugars). Similar growth rates on glucose and GlcNAc resembles the situation in *E. coli* [5] and it is interesting to correlate this rapid growth on GlcNAc with the *nagE* gene of *B. megaterium,* which encodes a three-domain EIICBA transporter, as does the *E. coli nagE* gene. Although the efficiency of use of GlcNAc appears to vary considerably in the different *Bacillaceae* tested, it seems clear that the absence of *gamAP* operon and the presence of just the *nagAB yvoA* and *nagP* genes is linked to preferential use of GlcNAc by the other *Bacillaceae*. This raises the question of why the *gamAP* operon has been acquired and maintained by *B. subtilis*. What ecological niche favours the use of amino sugars? Chitin, the polymer of N-acetylglucosamine, is the major source of amino sugars in nature and it is found in soil, the habitat of *B. subtilis,* derived from insect exoskeletons and fungal cell walls. Many organisms, including some *Bacilli* [48]–[50] secrete chitinases, which break down chitin to chitobiose, chitotriose etc and free GlcNAc. There are limited amounts of deacetylated residues in chitin but several fungi and insects encode chitin deacetylases [51], which might increase the amount of free GlcN liberated by chintinases. Moreover *B. subtilis* and other *bacilli* are common species found in the rhizosphere [52], which could be enriched for plant-derived amino sugars. Plant roots are a natural habitat for *B. subtilis* biofilm formation [53]. However why only *B. subtilis* of the variety of *Bacilli* found in the rhizosphere should have adapted to efficient use of GlcN is not obvious. Concerning use of amino sugars, *B. subtilis* cannot be considered as the model for Gram positive organisms.

## Supporting Information

Figure S1 **Mapping of the transcription start point of the** ***nag*** **and** ***gam*** **gene mRNAs.** The 5′ ends of the genes indicated were mapped by primer extension on RNA isolated from bacteria grown on Glc, GlcN or GlcNAc and using the oligonucleotides listed in Table S2. The same oligonucleotides were used to generate sequencing reactions (shown to the right). The putative −35, −10 sequences and position of the +1 are indicated (see also Table 2). The 5′ end of *nagP* was estimated based on the migration position of DNA markers (pBR322 digested with MspI).
(TIF)

Figure S2 **Synteny of the genes involved in utilization of GlcN and GlcNAc.** The genomic contexts, spanning 15 kb, of the various genes involved in amino sugar catabolism in *Bacillaceae* were analyzed with the SyntTax web server (Oberto, 2013), using as query sequences the corresponding proteins originating from *Bacillus subtilis*. The following genes were considered: *nagA* (A), *nagB* (B), *nagP* (C), *ptsG* (D), *yvoA(nagR)* (E), *ypqE* (F), *gamA* (G), *gamP* (H), *ybgA* (I) and *ybgB* (J). The gene color code is consistent for each individual synteny analysis and the gene corresponding to the query protein is represented with an arrow outlined in bold. The genes are labeled according to the original NCBI annotations and were not corrected for consistency. (Note the *gamA* gene of *B. subtilis* 168 has been annotated as *nagBB*, the *gamP* gene as a second *nagP* and *nagB* called *nagBA*.) For clarity reasons, only one exemplar of each species was included in this analysis. In each subfigure, the genomes are ranked by the normalized Blast score, obtained by matching the query protein to the selected chromosomes. This score reflects percentage orthology and is computed as described [56]. Values of less than 100% for the starting protein in some genomic contexts are caused by the presence of repetitive/low complexity sequences, which are ignored by the initial TBlastN alignments. Sporadic color conservation among unrelated genes can be observed in genomes with low scores and can be explained by the presence of shared transport/transmembrane domains in the corresponding proteins. Comparing the A to J subfigures shows that the *nagA*-*nagB*-*yvoA* operon structure is maintained as a rather stable synton throughout the *Bacillaceae*. The gradual decrease in score reflects the natural genetic drift observed in orthologous proteins. Conservation of the *ybgB*-*ybgA*-*gamA*-*gamP* synton is extremely limited and only visible in *Bacillus subtilis* and its close relatives such as *B. atropheus*. This synton has not been found so far in any other sequenced prokaryote. The abrupt decrease in score for these four factors in organisms below *B. atropheus* strongly suggests that the corresponding genes were acquired by lateral gene transfer.
(PDF)

Table S1 **% identities of** ***B. subtilis nag*** **and** ***gam*** **proteins.** *Bacillus subtilis* proteins unless stated otherwise. Alternative nomenclature is given in brackets. BLASTp scores are shown as number of identical amino acids over the longest alignment and relative percentage. NS: Not significant.
(PDF)

Table S2 **Oligonucleotides used.** Restriction sites introduced into oligonucleotides are underlined. ^1^A and AT were introduced at the 5′ end of ps1 and ps4 (Datsenko KA, Wanner BL (2000) Proc Natl Acad Sci USA 97: 6640–6645) to ensure that the scar sequence after elimination of the *spec* cassette forms an in-phase peptide with the start and stop codons of the replaced gene.
(PDF)

Table S3 **Doubling times of the** ***gam*** **and** ***nag*** **mutants of** ***B. subtilis*** **during growth on GlcN and GlcNAc.** Growth rates are given as doubling times (min) of the different *nag* and *gam* mutants during growth on GlcN and GlcNAc. The alternative name for *yvoA* is *nagR* [9]. DT were calculated by regression analysis of log OD versus time, generally in range 0.1–1.5 OD. Values are the mean doubling times (± standard deviation) of two to four cultures. NG = no growth. These are the data used to make Figure 5 and mutant strains are numbered as in that figure.
(PDF)
