# Supplementary figures and images for: The Use of Amino Sugars by Bacillus subtilis: Presence of a Unique Operon for the Catabolism of Glucosamine
Source: PLoS One. 2013 May 8;8(5):e63025. doi: 10.1371/journal.pone.0063025 (PMC3648570; doi:10.1371/journal.pone.0063025)

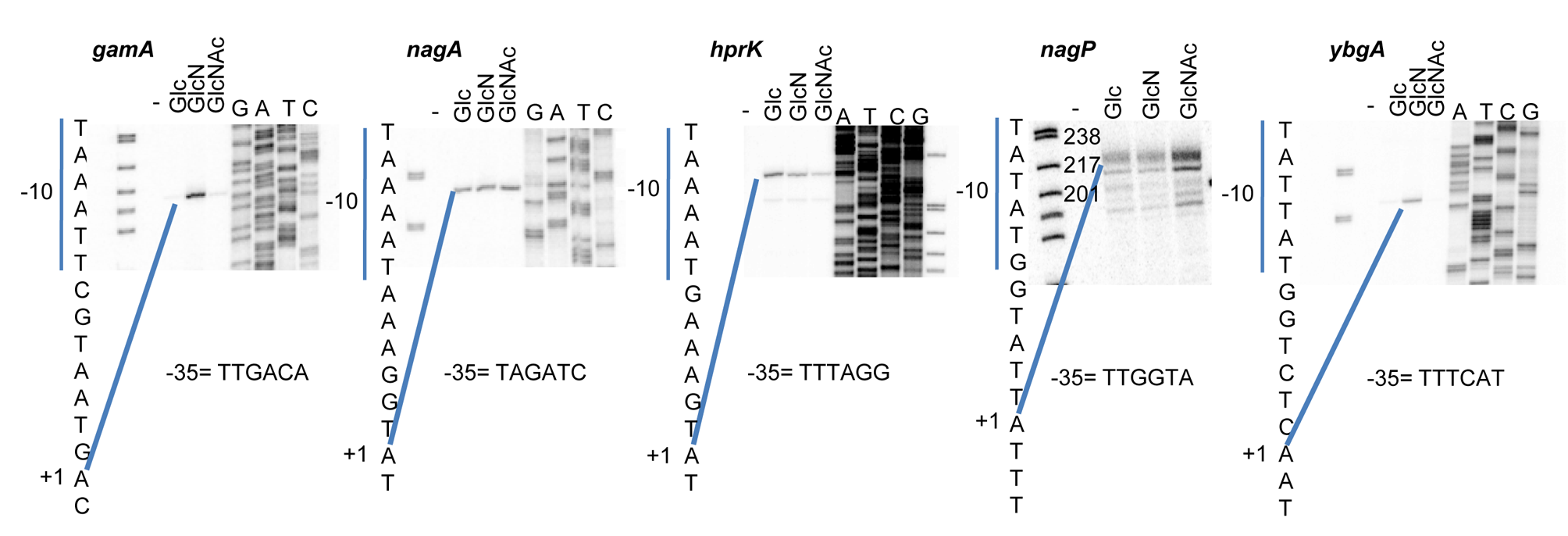

Supplement: Figure S1 — Mapping of the transcription start point of the nag and gam gene mRNAs. The 5′ ends of the genes indicated were mapped by primer extension on RNA isolated from bacteria grown on Glc, GlcN or GlcNAc and using the oligonucleotides listed in Table S2. The same oligonucleotides were used to generate sequencing reactions (shown to the right). The putative −35, −10 sequences and position of the +1 are indicated (see also Table 2). The 5′ end of nagP was estimated based on the migration position of DNA markers (pBR322 digested with MspI). (TIF) [file pone.0063025.s001.tif]
