# Supplementary material for: The Use of Amino Sugars by Bacillus subtilis: Presence of a Unique Operon for the Catabolism of Glucosamine
Source: PLoS One. 2013 May 8;8(5):e63025. doi: 10.1371/journal.pone.0063025 (PMC3648570; doi:10.1371/journal.pone.0063025)

Figure S2 A. *Bacillaceae nagA* synteny

Genomic contexts

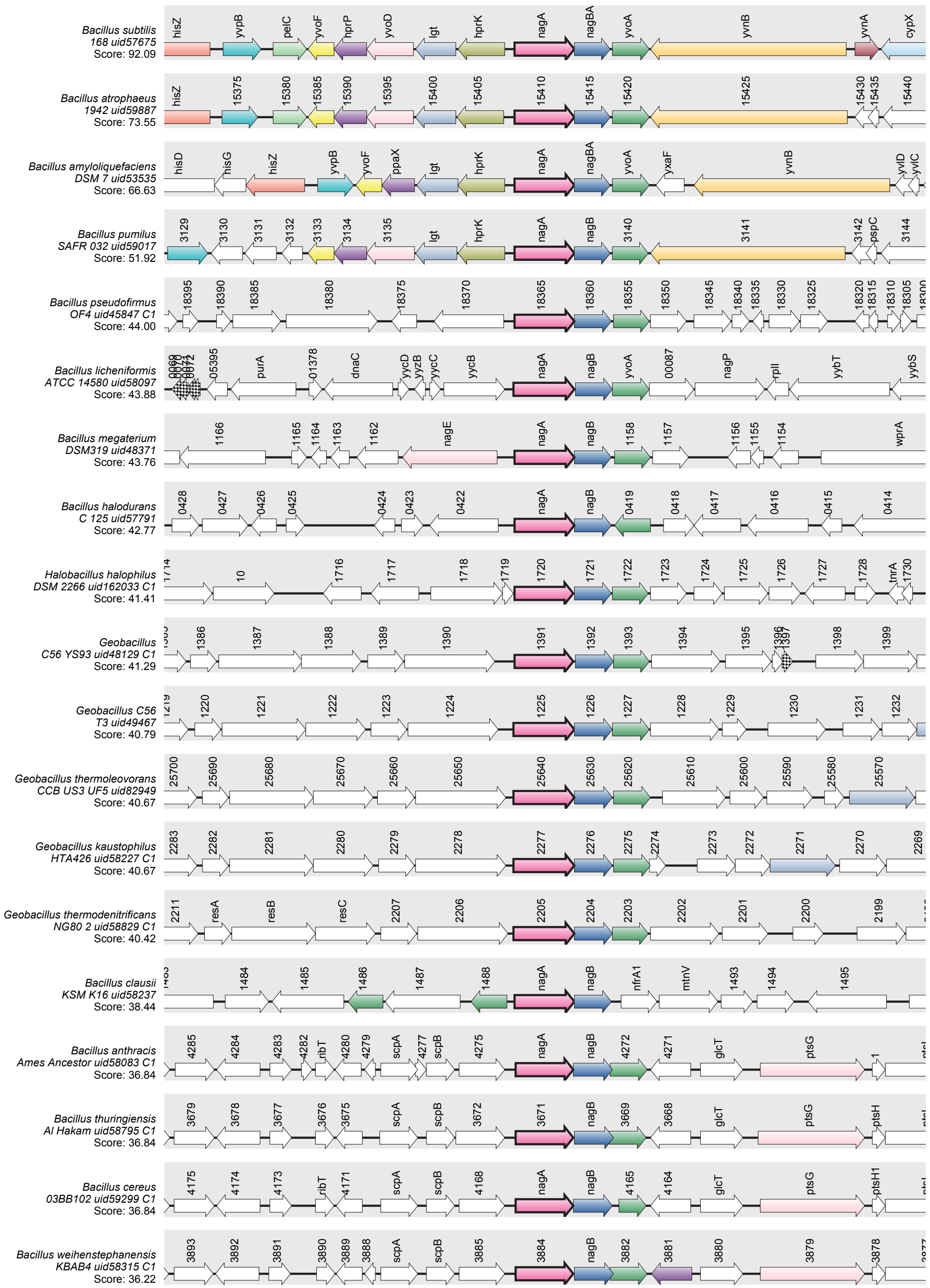

SyntTax Report

http://archaea.u-psud.fr/SyntTax

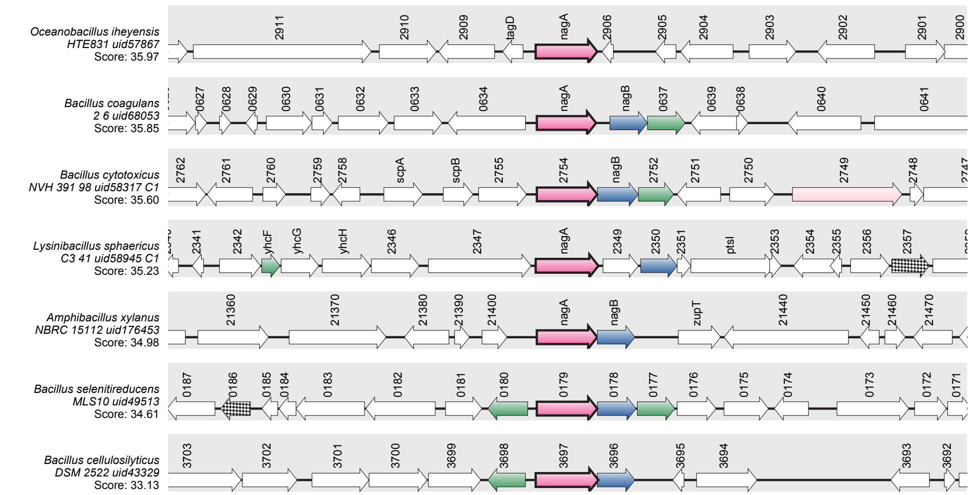

Figure S2 B. *Bacillaceae nagB* synteny

Genomic contexts

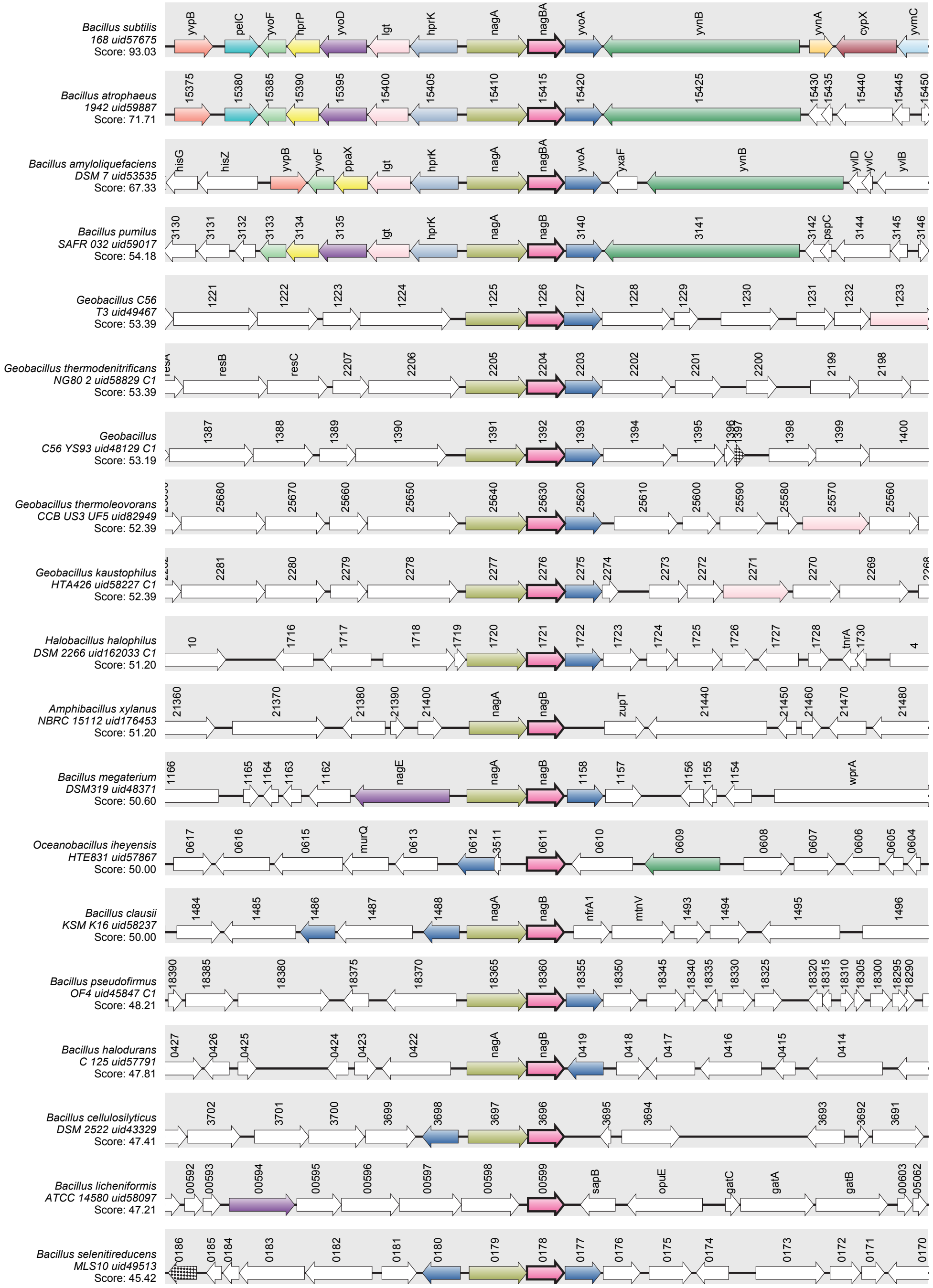

SyntTax Report

http://archaea.u-psud.fr/SyntTax

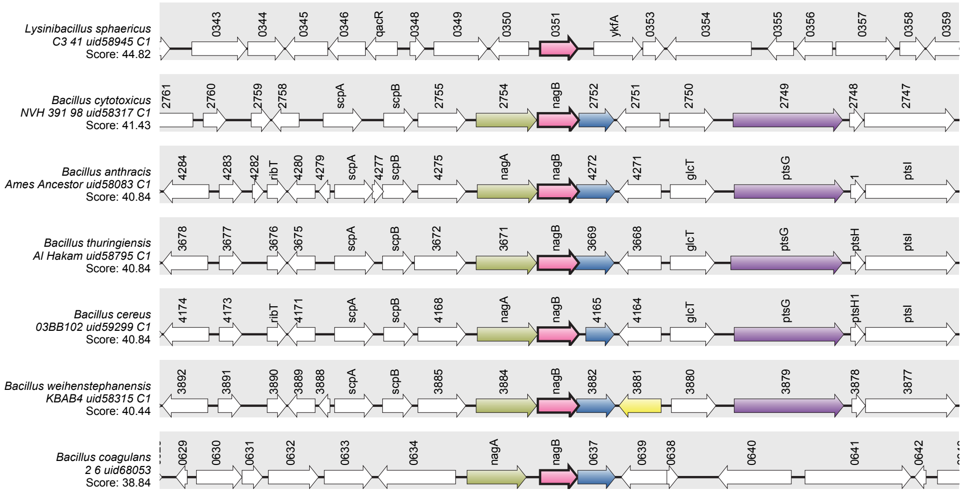

Figure S2 C. *Bacillaceae nagP* synteny

Genomic contexts

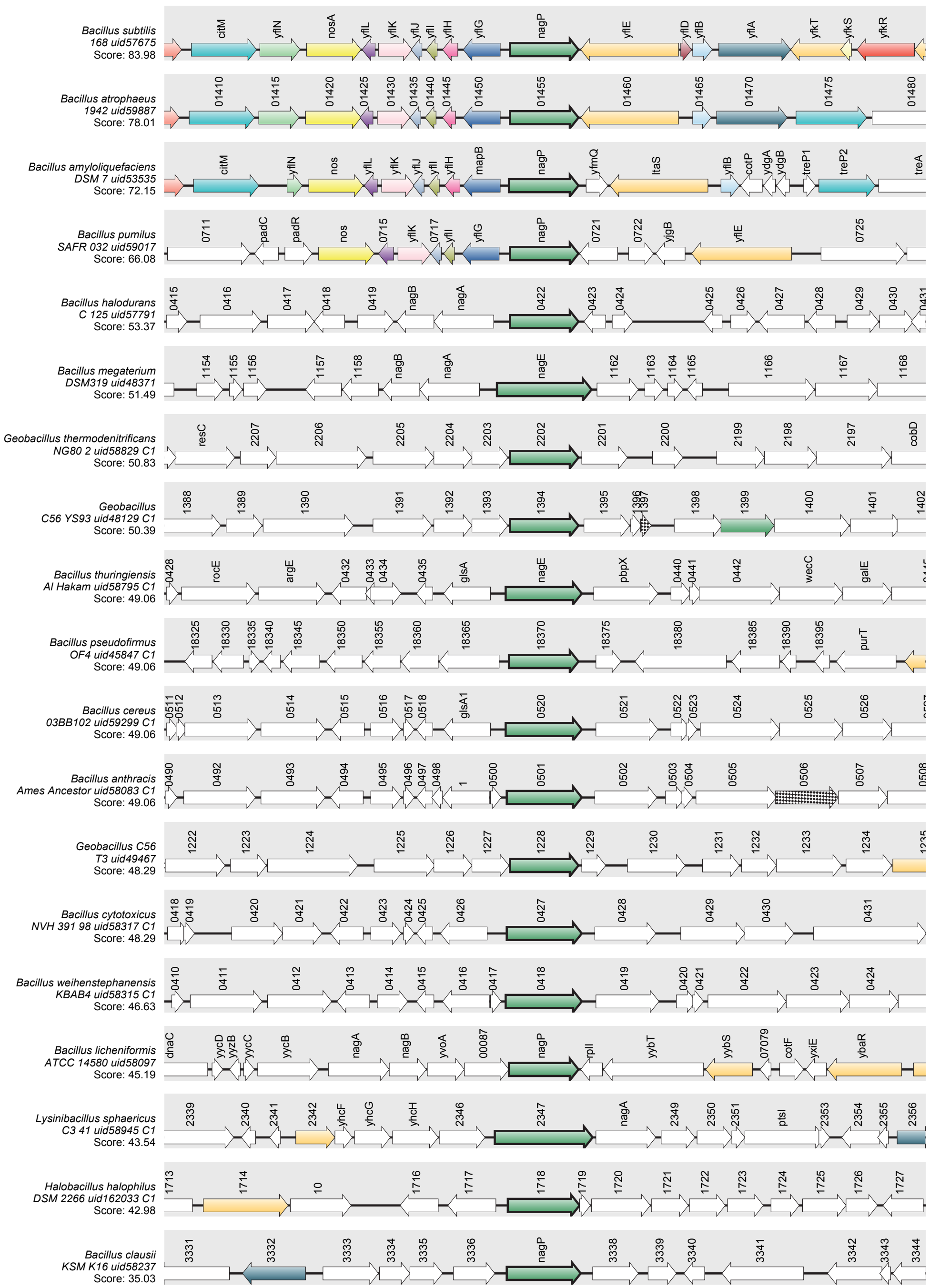

SyntTax Report

http://archaea.u-psud.fr/SyntTax

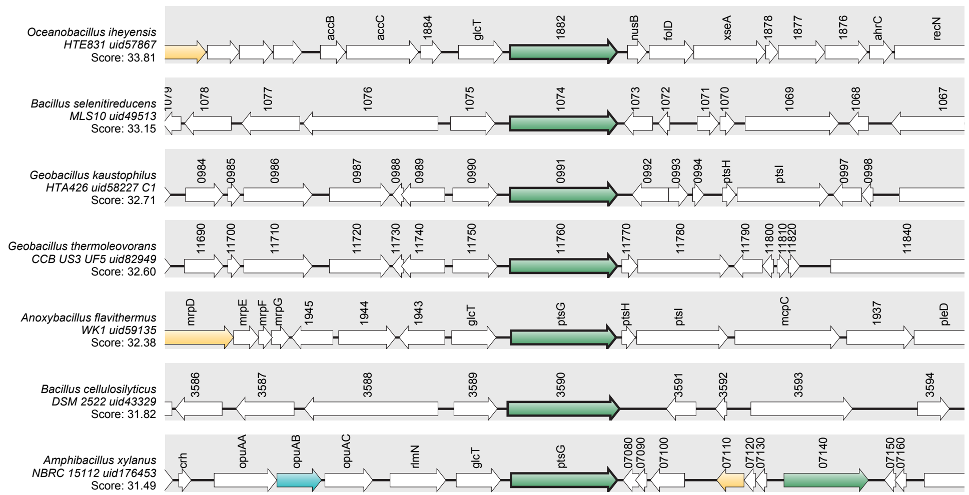

Figure S2 D. *Bacillaceae ptsG* synteny

Genomic contexts

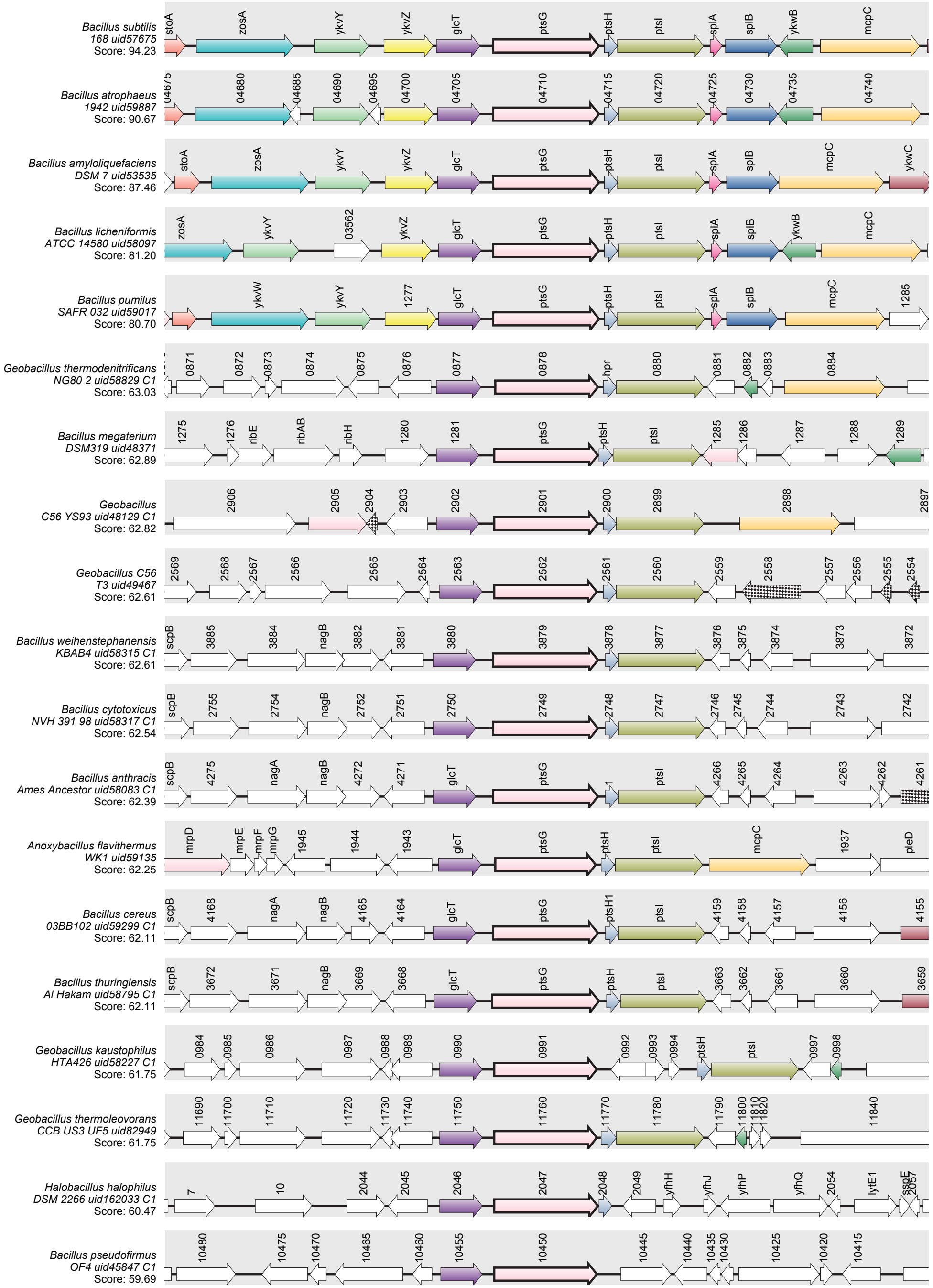

SyntTax Report http://archaea.u-psud.fr/SyntTax

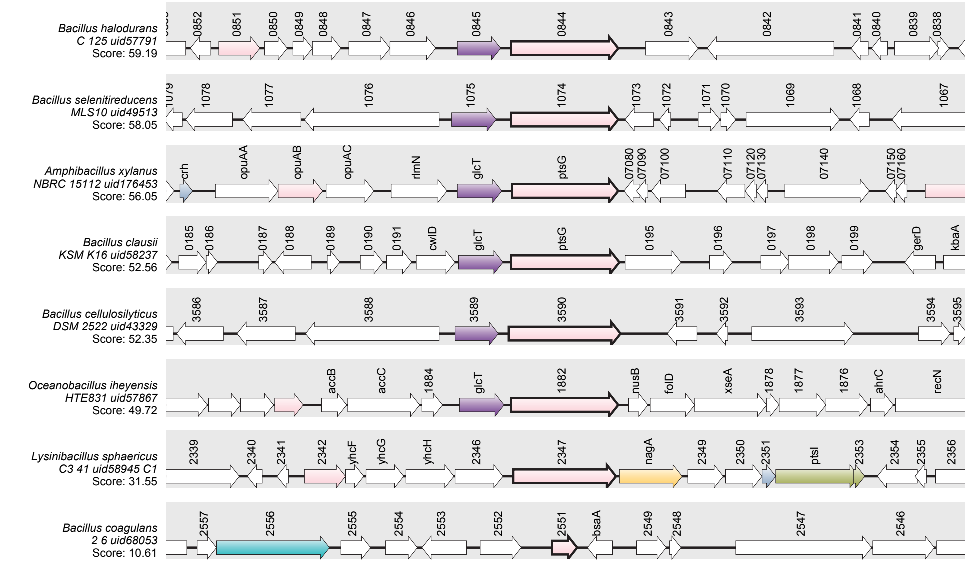

Figure S2 E. *Bacillaceae ypqE* synteny

Genomic contexts

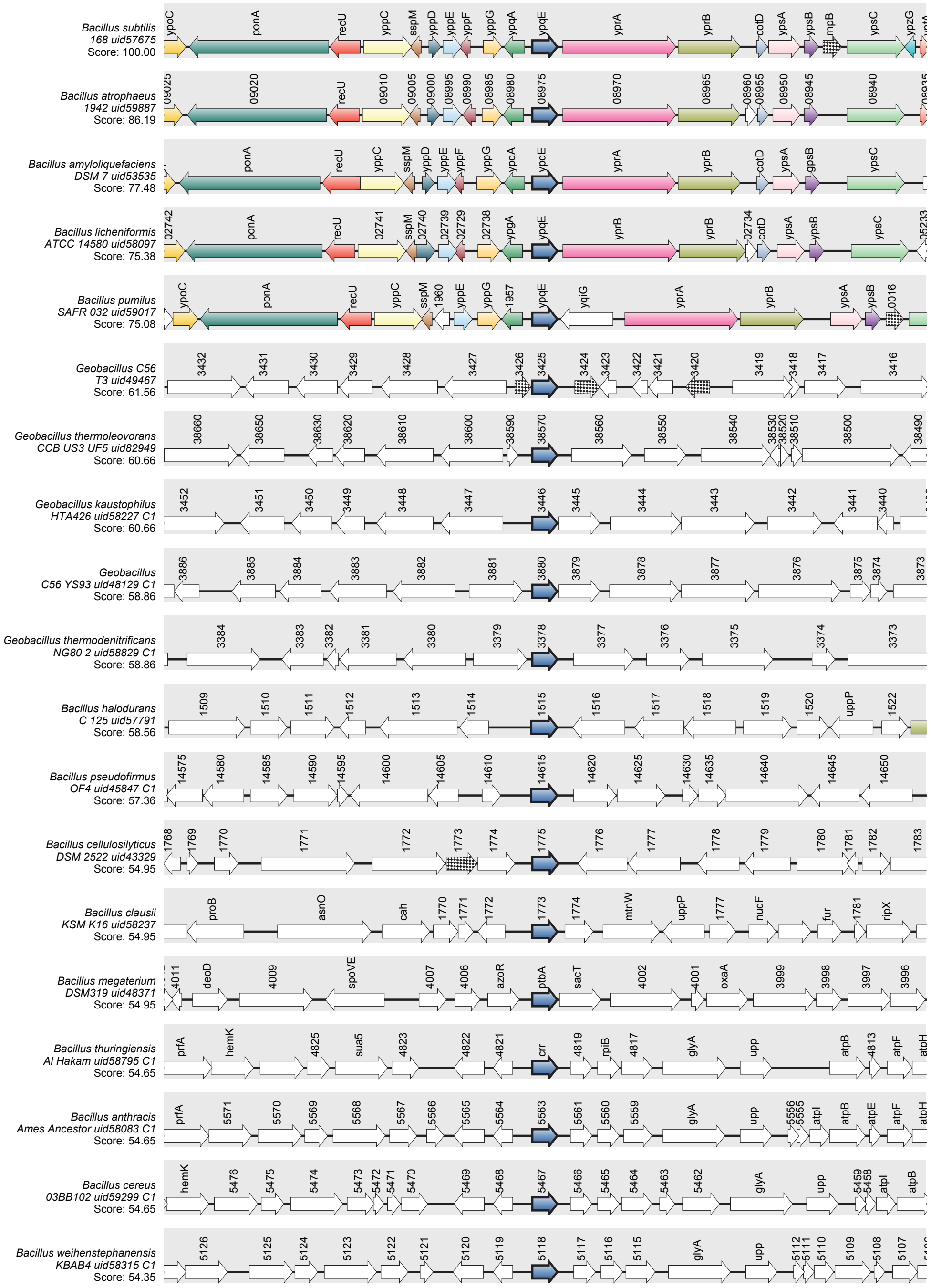

SyntTax Report

http://archaea.u-psud.fr/SyntTax

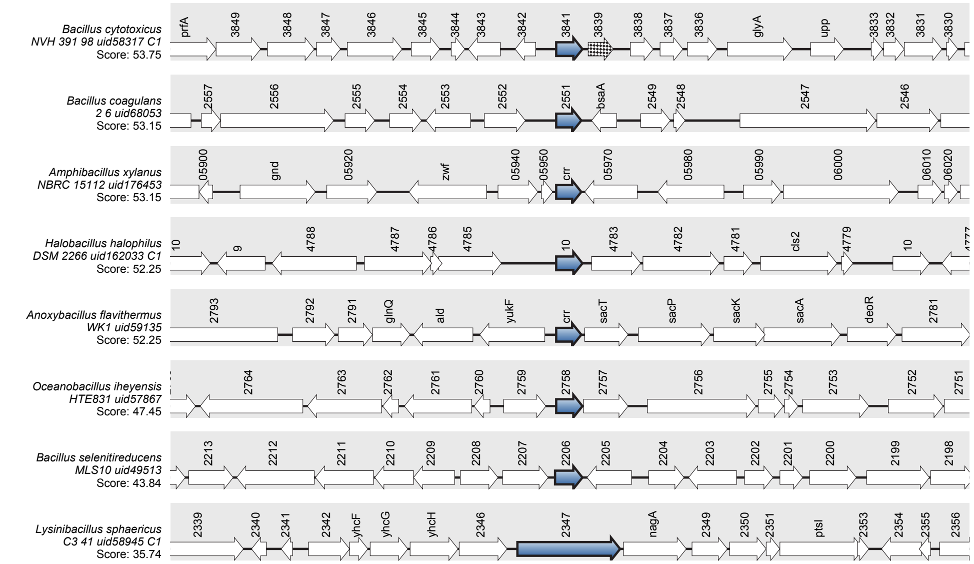

Genomic contexts

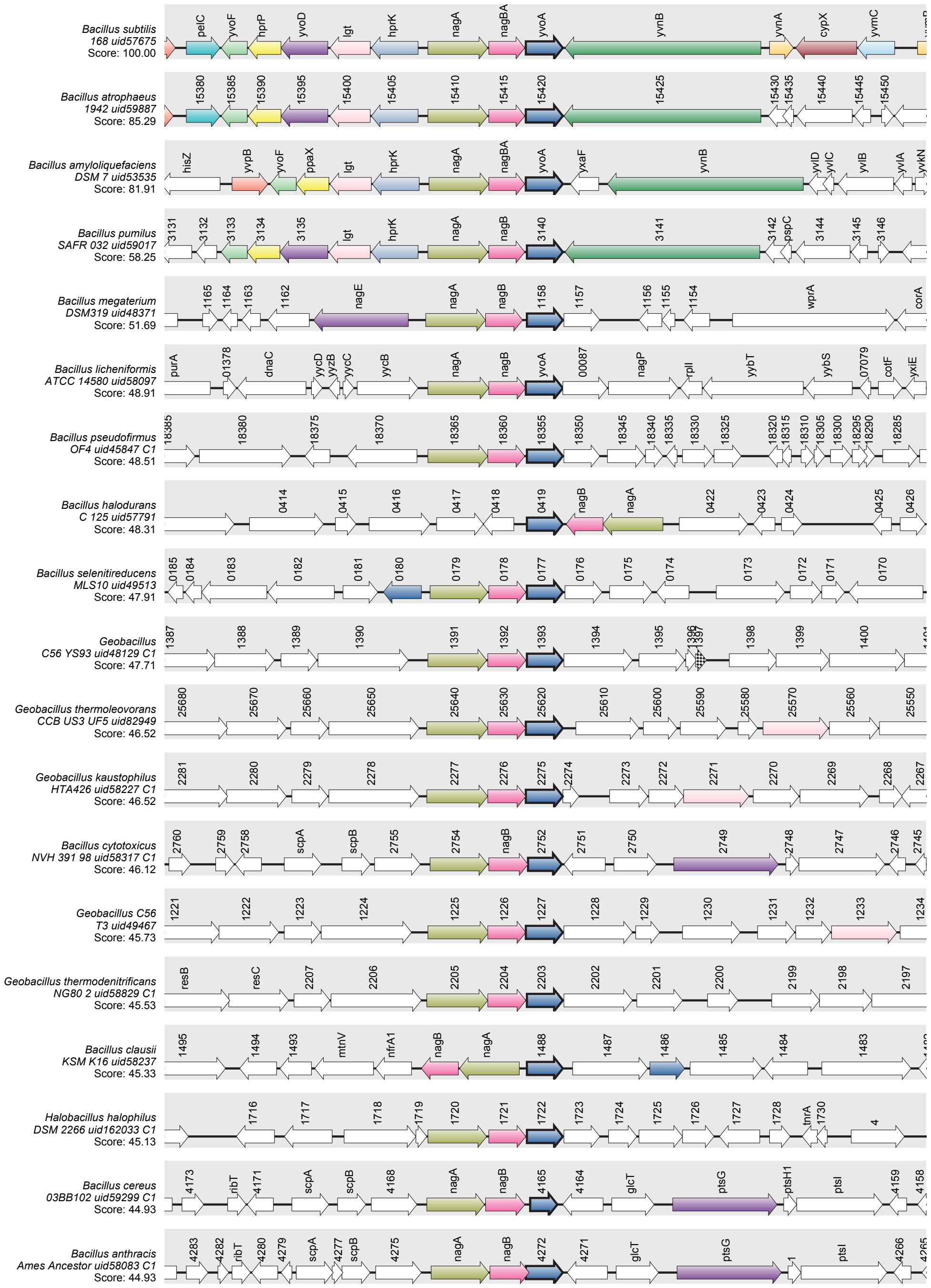

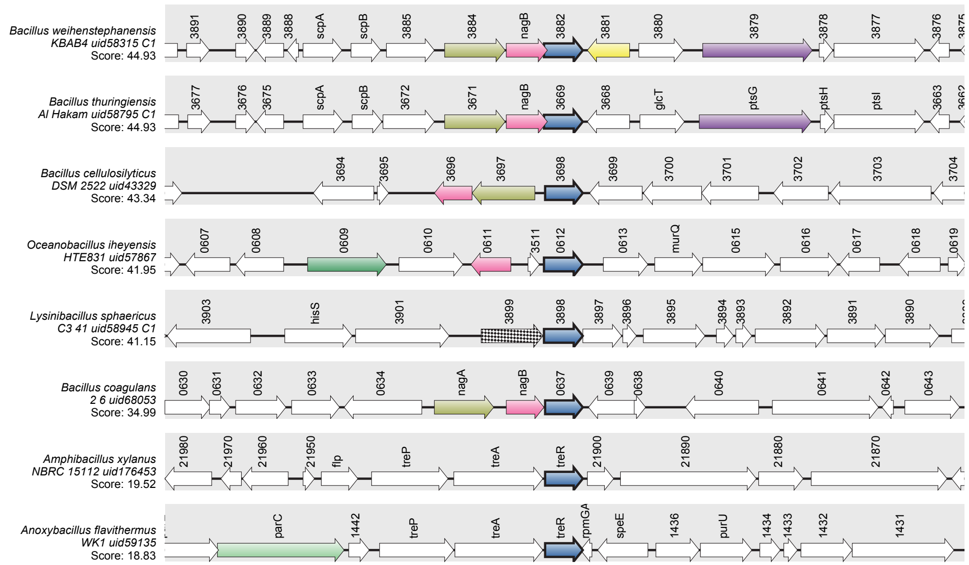

Figure S2 G. *Bacillaceae gamA* synteny.

Genomic contexts

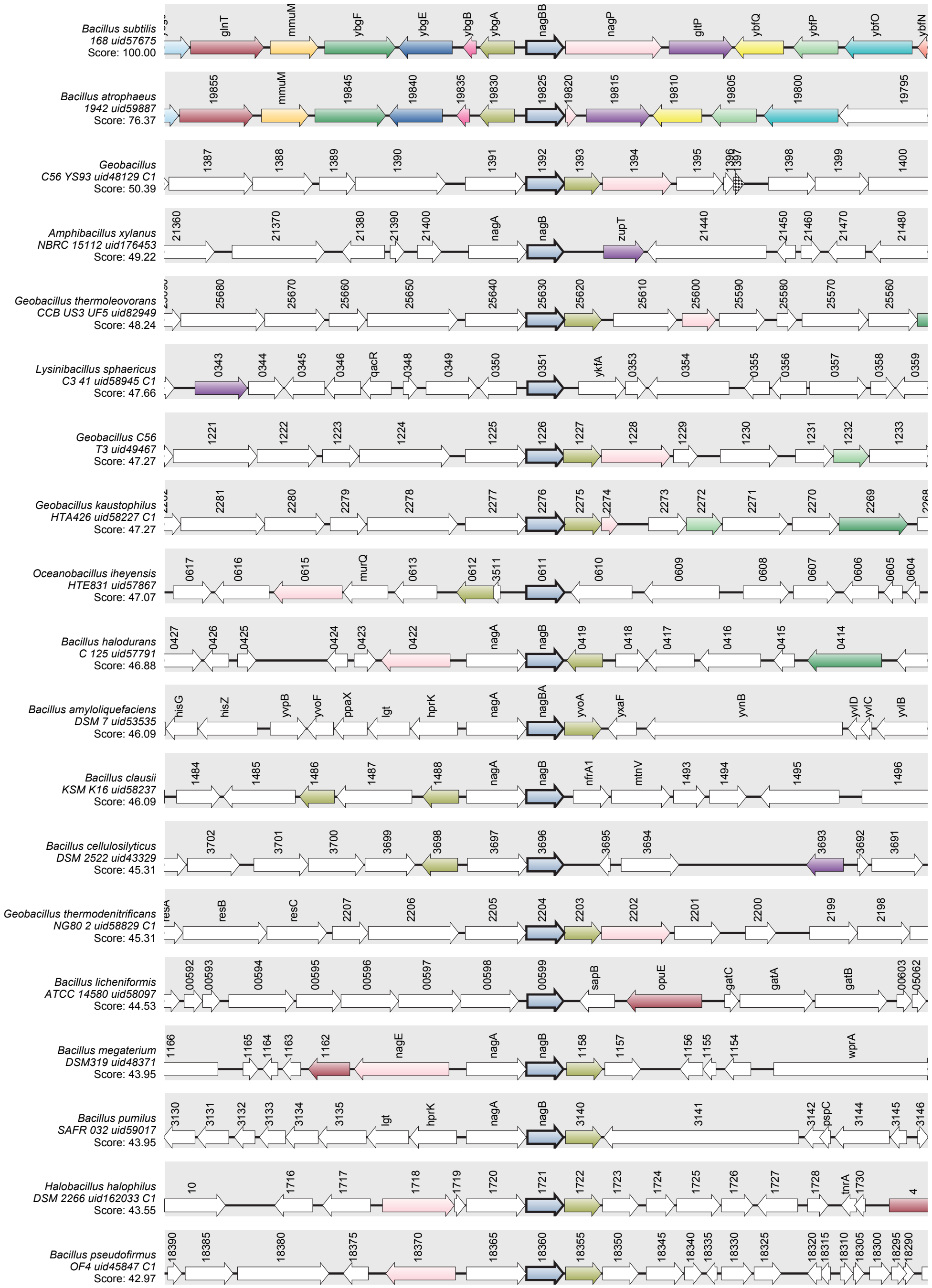

SyntTax Report http://archaea.u-psud.fr/SyntTax

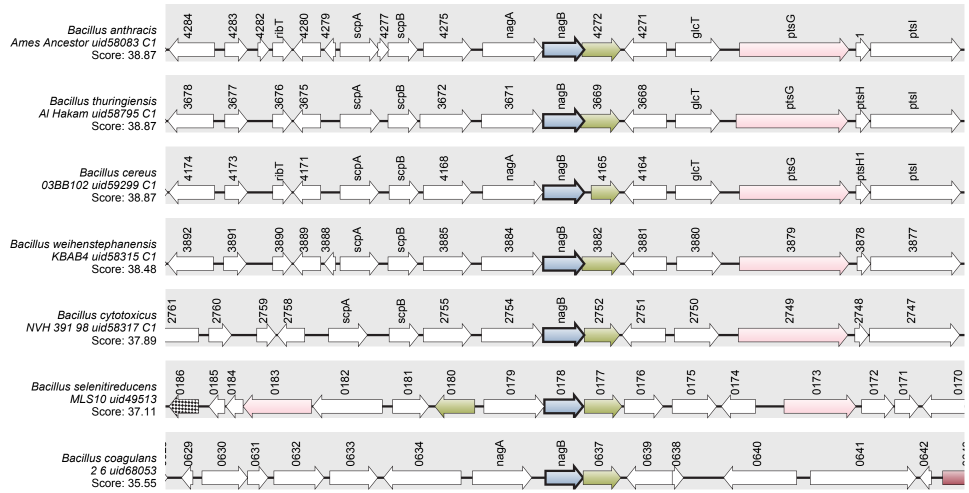

Figure S2 H. *Bacillaceae gamP* synteny

Genomic contexts

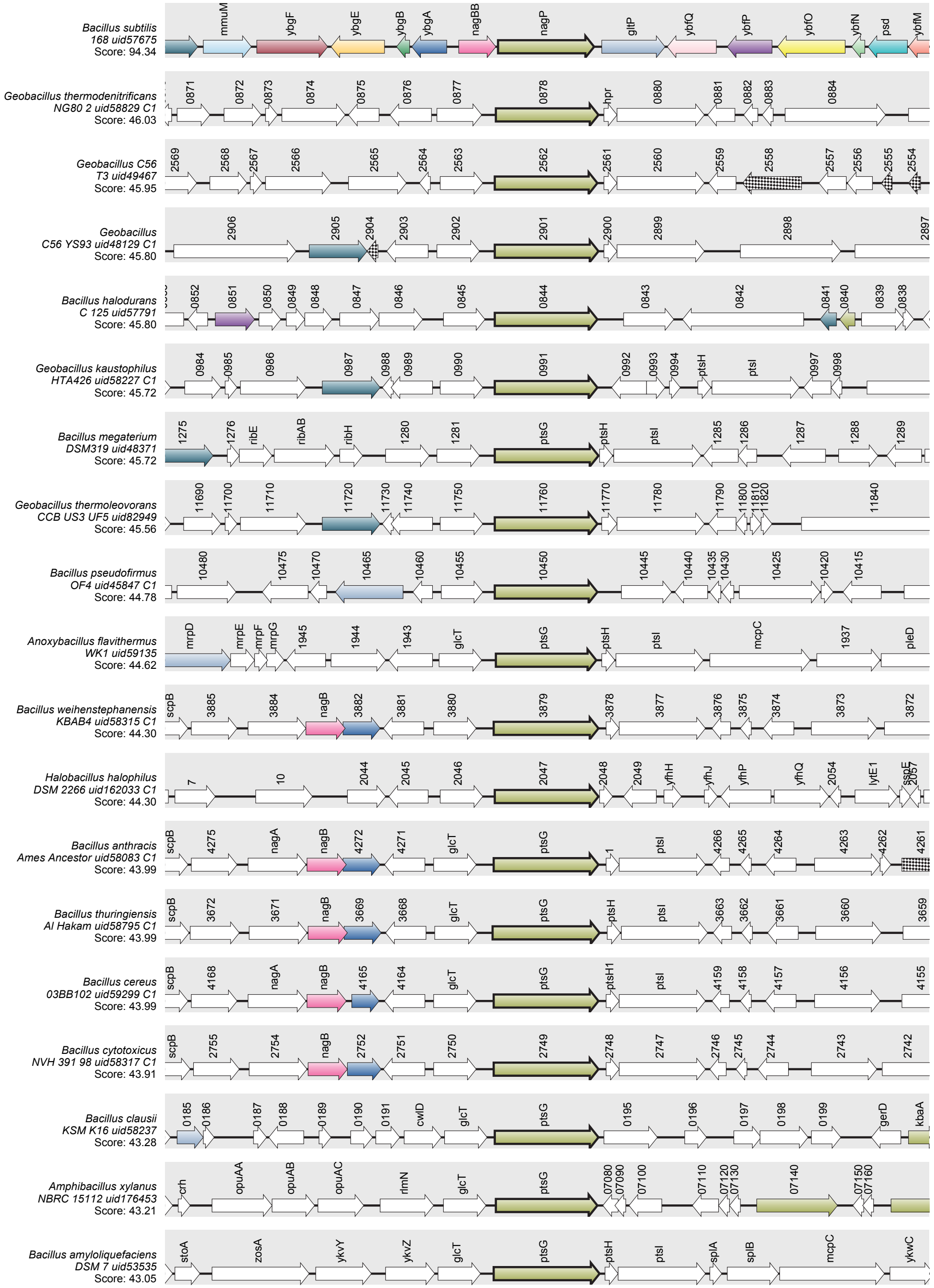

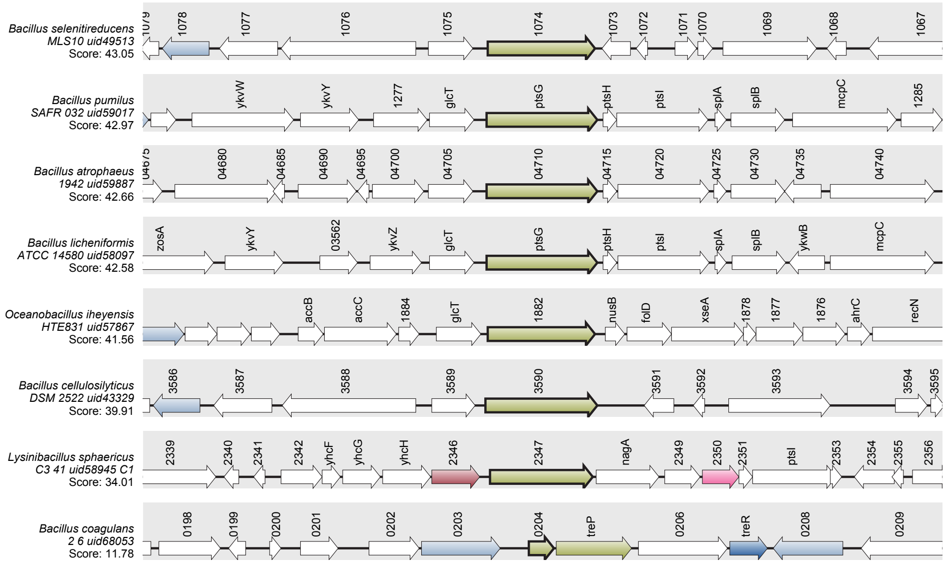

Genomic contexts

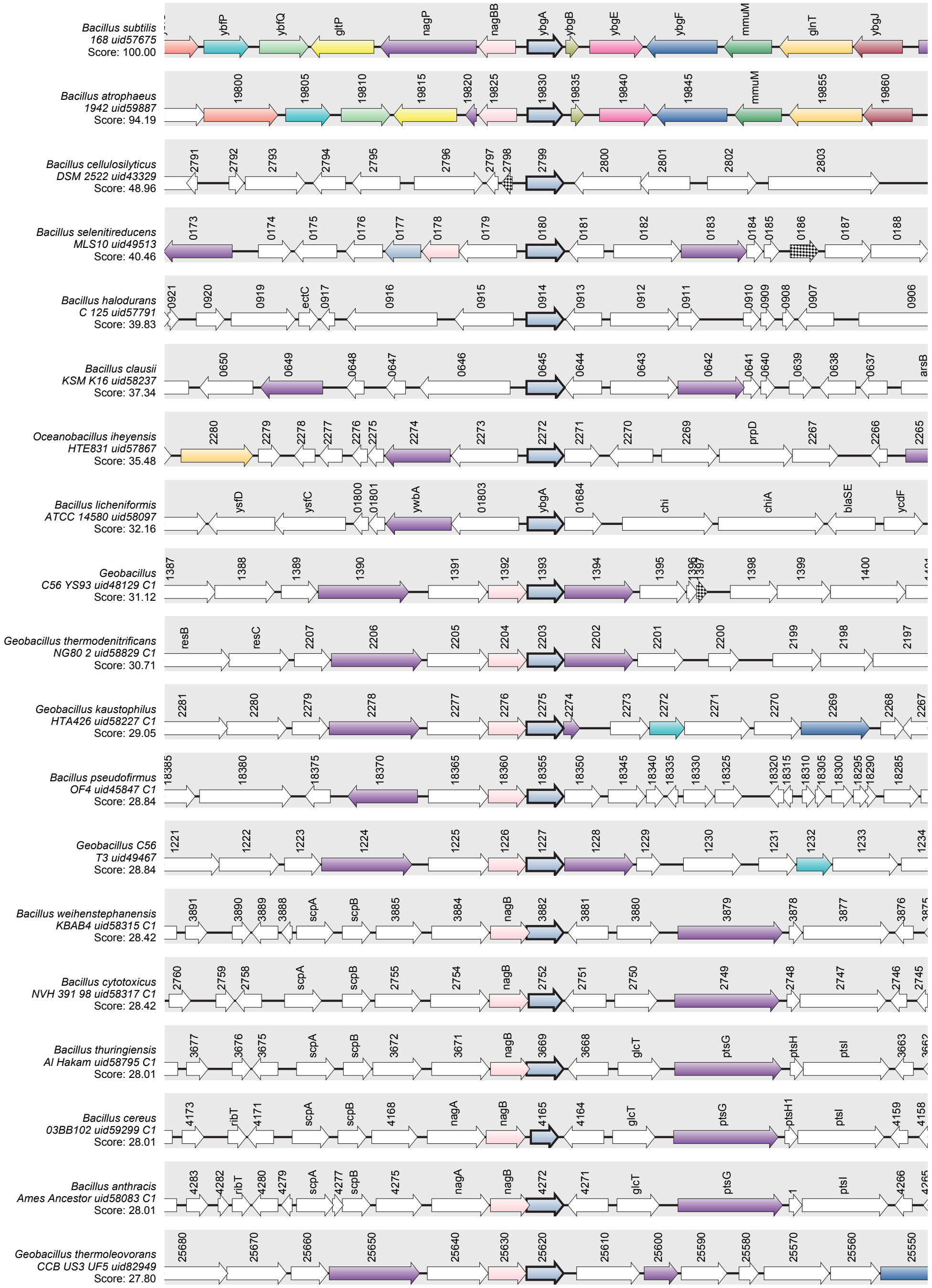

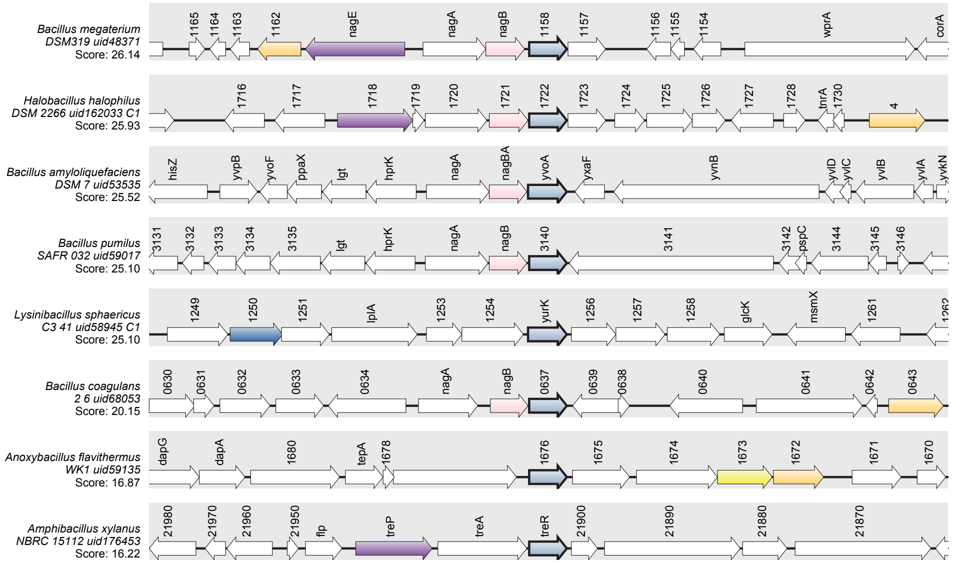

Genomic contexts

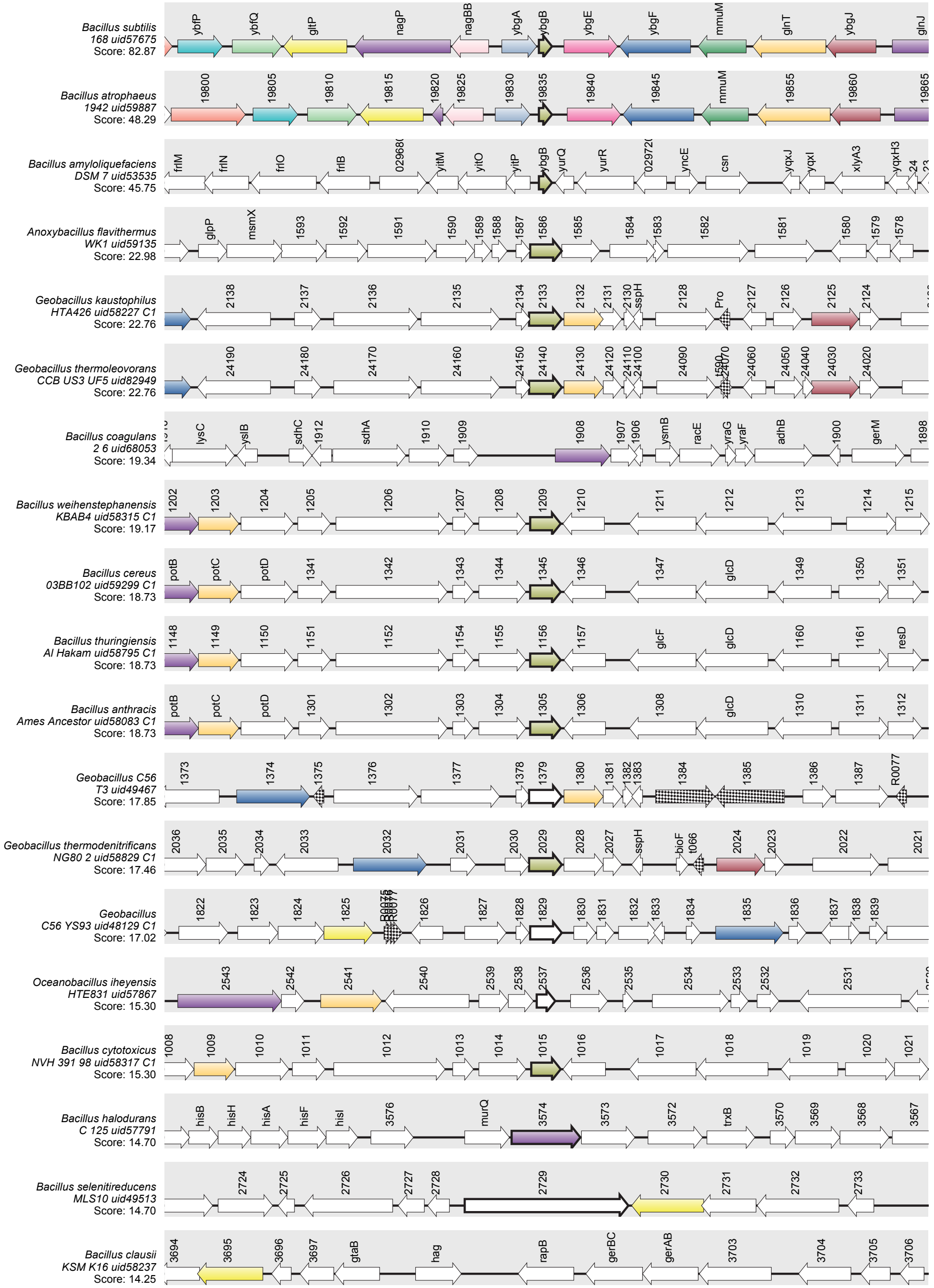

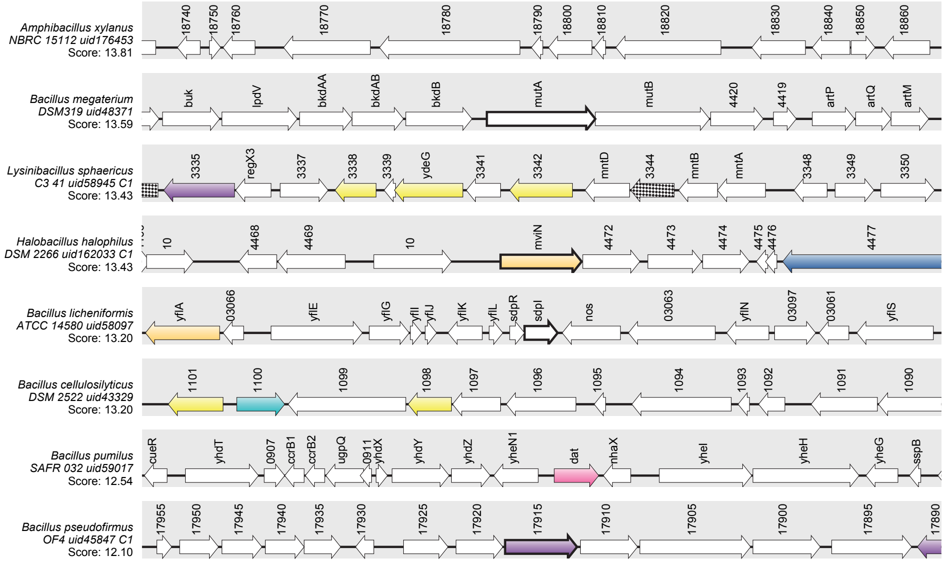

Supplement: Figure S2 — Synteny of the genes involved in utilization of GlcN and GlcNAc. The genomic contexts, spanning 15 kb, of the various genes involved in amino sugar catabolism in Bacillaceae were analyzed with the SyntTax web server (Oberto, 2013), using as query sequences the corresponding proteins originating from Bacillus subtilis. The following genes were considered: nagA (A), nagB (B), nagP (C), ptsG (D), yvoA(nagR) (E), ypqE (F), gamA (G), gamP (H), ybgA (I) and ybgB (J). The gene color code is consistent for each individual synteny analysis and the gene corresponding to the query protein is represented with an arrow outlined in bold. The genes are labeled according to the original NCBI annotations and were not corrected for consistency. (Note the gamA gene of B. subtilis 168 has been annotated as nagBB, the gamP gene as a second nagP and nagB called nagBA.) For clarity reasons, only one exemplar of each species was included in this analysis. In each subfigure, the genomes are ranked by the normalized Blast score, obtained by matching the query protein to the selected chromosomes. This score reflects percentage orthology and is computed as described [56]. Values of less than 100% for the starting protein in some genomic contexts are caused by the presence of repetitive/low complexity sequences, which are ignored by the initial TBlastN alignments. Sporadic color conservation among unrelated genes can be observed in genomes with low scores and can be explained by the presence of shared transport/transmembrane domains in the corresponding proteins. Comparing the A to J subfigures shows that the nagA-nagB-yvoA operon structure is maintained as a rather stable synton throughout the Bacillaceae. The gradual decrease in score reflects the natural genetic drift observed in orthologous proteins. Conservation of the ybgB-ybgA-gamA-gamP synton is extremely limited and only visible in Bacillus subtilis and its close relatives such as B. atropheus. This synton has not been found so far in [file pone.0063025.s002.pdf]
