# Supplementary material for: The Use of Amino Sugars by Bacillus subtilis: Presence of a Unique Operon for the Catabolism of Glucosamine
Source: PLoS One. 2013 May 8;8(5):e63025. doi: 10.1371/journal.pone.0063025 (PMC3648570; doi:10.1371/journal.pone.0063025)
Supplement: Table S1 — % identities of B. subtilis nag and gam proteins. Bacillus subtilis proteins unless stated otherwise. Alternative nomenclature is given in brackets. BLASTp scores are shown as number of identical amino acids over the longest alignment and relative percentage. NS: Not significant. (PDF) [file pone.0063025.s003.pdf]

Table S1. % identities of *B. subtilis gam* and *nag* proteins

YbgA/YvoA (NagR)<br>72/214<br>34%

	**GamA** (YbfT,NagBB) 249aa	**GamP** (YbfS,NagP) 631aa	**NagA** 396aa	**NagB** (NagBA) 242aa	**NagP** (YflF) 452aa	**NagE** (*E. coli*) 648aa	**NagB** (*E.coli*) 266aa	**NagA** (*E. coli*) 382aa	**YpqE** 168aa	**PtsG** 687aa
GamA (YbfT,NagBB)		NS	NS	123/242 51%	NS	NS	93/249 37%	NS	NS	NS
GamP (YbfS, NagP)			NS	NS	198/467 42%	267/632 42%	NS	NS	63/125 50%	346/687 50%
NagA				NS	NS	NS	NS	120/380 32%	NS	NS
NagB (NagBA)					NS	NS	95/240 40%	NS	NS	NS
NagP (YflF)						234/465 50%	NS	NS	NS	200/500 40%
NagE (*E. coli*)							NS	NS	51/145 35%	273/702 39%
NagB (*E. coli*)								NS	NS	NS
NagA (*E. coli*)									NS	NS
YpqE										60/130 46%

Bacillus subtilis proteins unless stated otherwise. Alternative nomenclature is given in brackets.
BLASTp scores are shown as number of identical amino acids over the longest alignment and relative percentage. NS: Not significant.
