# Supplementary material for: The Use of Amino Sugars by Bacillus subtilis: Presence of a Unique Operon for the Catabolism of Glucosamine
Source: PLoS One. 2013 May 8;8(5):e63025. doi: 10.1371/journal.pone.0063025 (PMC3648570; doi:10.1371/journal.pone.0063025)
Supplement: Table S2 — Oligonucleotides used. Restriction sites introduced into oligonucleotides are underlined. 1A and AT were introduced at the 5′ end of ps1 and ps4 (Datsenko KA, Wanner BL (2000) Proc Natl Acad Sci USA 97: 6640–6645) to ensure that the scar sequence after elimination of the spec cassette forms an in-phase peptide with the start and stop codons of the replaced gene. (PDF) [file pone.0063025.s004.pdf]

Table S2. Oligonucleotides used

Oligonucleotide	Sequence	Fragment amplified or Function
**Cloning chromosomal regions of *B.subtilis***		
Gam4 BamHI	GTCAGGATCCGGACGGAACAGCAGCACC	4.8 kb fragment carrying *ybgAgamAP*
Gam5 XbaI	CTAGTCTAGAGGTCAAGCCCGACACCTGG	
BsNag1 BamHI	CTGAGGATCCTTCGAGTATCCCTTGACGCG	5.3 kb fragment carrying *hprK-nagAByvoA(nagR)*
BsNag2 XbaI	CTAGTCTAGACATCGCGTGGATGAATCAGG	
BsNag4	CGAACCTTTGCCACGATATG	2.9 kb fragment carrying *nagAByvoA(nagR)*
BsNag8	AGAAACATATCGTCCGGCAC	
5'NagP NcoI	CTGACCATGGAAGGCGTTCTTCTCCTTCAC	2.6 kb fragment carrying *nagP*
3'NagP BamHI	GTCAGGATCCAGTCAGCCCATTACCTTG	
ptsG2 XbaI	GACTTCTAGACATTGCGTTGACAGACCATATCGC	3.6 kb fragment carrying *ptsGHI*
ptsG 4 BamHI	AAGCGGATCCAGCGTTCACTGAATCAGTGCTG	
YpqE1 XbaI	GCCATCTAGAAACGTCGCCGGAATGGT	1.5 kb fragment carrying *ypqE*
YpqE2 SphI	GTATGCATGCGGTAGAATGGCAGAATGCAGC	
5' FLP AflII	GATCCTTAAGCTAACACCGTGCGTGTTGAC	1.5 kb fragment carrying *flp* from pCP20
3'FLP SbfI	CAGTCCTGCAGGCACGTCGCACCTATATCTGC	
**Spec cassette with 50 bp targeting sequences**		
5'NagA ps4	GTCTGAAAAAGAGTAAAATAAAGGTATTCAAATTCCAGA AAGGCGGATCATCTATGTTCCGGGGATCCGTCGACC	ps4-ps1spec cassette with *nagA* targeting sequences
3'NagA ps1	TTCCATTACTTTCATATCTGGTCAGCCTCCTTGGATATA AATGCAATGTTGTGTAGGCTGGAGCTGCTTC	
5'NagB ps4	CACCATTTGCCGCGGAAACATTGCATTTATATCCAAGGA GGCTGACCAGATATGATTCCGGGGATCCGTCGACC	ps4-ps1spec cassette with *nagB* targeting sequences
3'NagB ps1	ATTGATATTCATAAGTCAGCATGTTCCTTTCAAGGTCTT AATGACGCGGCGTGTAGGCTGGAGCTGCTTC	
5'yvoA ps4	GATTGATCGTGAAGCCGCGTCATTAAGACCTTGAAAGGA ACATGCTGACTTATGATTCCGGGGATCCGTCGACC	ps4-ps1spec cassette with *yvoA (nagR*) targeting sequences
3'yvoA ps1	GAAAGACGATCCATATAGTGGACAAATGTATAACGGTCG CCTCTGTATACGGAATGTGTAGGCTGGAGCTGCTTC	
5'NagP ps4	CACTAGATATCTTATATGGTATATTTGAAAAAAAAGGGT ATGAGGGGGATGGGTATGATTCCGGGGATCCGTCGACC	ps4-ps1spec cassette with *nagP* targeting sequences
3'NagP ps1	GCGGAGAGGTTGCCCTCTCCGCTTTTTTATTTGACAGCA GCCCTCAATTCGTGTAGGCTGGAGCTGCTTC	
5'GamA ps4	GGTCATAACAAATATGGTGCTTGTCTATCTCTTTGAGGG GGTGTCACATGTTCCGGGGATCCGTCGACC	ps4-ps1spec cassette with *gamA* targeting sequences
3'GamA ps1	ATGCCTTTTTAAACATGACAGTCTCCTTTTATTGTGTTA TGAGAATGATGCACTATGTGTAGGCTGGAGCTGCTTC	
5'GamP ps4	GCTCAAAAATTAAAGAGTGCATCATTCTCATAACACAAT AAAAGGAGACTGTCATGATTCCGGGGATCCGTCGACC	ps4-ps1spec cassette with *gamP* targeting sequences
3'GamP ps1	CGAAAAAGTCCCCCGCAGCAGGGGGGACTTTTTTACTTC ATTTGAATGTGCTTGTGTAGGCTGGAGCTGCTTC	
5'ybgA ps4	GGTATTATGGTCTCAATGAAAAAGAACGGATTGCATACA GAATGGGGAGAATGAAATGATTCCGGGGATCCGTCGACC	ps4-ps1spec cassette with *ybgA* targeting sequences
3'ybgA ps1	GATATGACTGTTCTACGGTGAATTTCGTTCGGTCTCCCC GTGTAATAATTTGCGCATGTGTAGGCTGGAGCTGCTTC	
5' ptsG ps4	GATTTTTAGCTGTAAGGTCAGACTAGTAAAAAGAGGAGG TCAATTCTTATGATTCCGGGGATCCGTCGACC	ps4-ps1spec cassette with *ptsG* targeting sequences
3' ptsG ps1	CATCTGACAAGCACGGCGTACTAACACCCTTATTTTTCA ATCTTCACAATATGTGTAGGCTGGAGCTGCTTC	

5'ypqEps4	GTGAATGAAGGGATAACGGTTGAAGTGAATGAGAAGGG AGAAAATACATTGATTCCGGGGATCCGTCGACC	ps4-ps1spec cassette with *ypqE* targeting sequences
3'ypqEps1	GAATAAAAAAAGCAAGGCATACGCCCTGCTTATTTCGCT TTTATTGTAAAATGTGTAGGCTGGAGCTGCTTC	
5' kanspec	AGAGCGCTTTTGAAGCTCACGCTGCCGCAAGCACTCAGG GCGCAAGGGCTAAGCTTGATATCGAATTCCTGCAGC	1,5 kb fragment carrying *spec* with *kan* targeting sequences
3' kanspec	GAATAGGAACTTCAAGATCCCCTTATTAGAAGAACTCGT CAAGAAGGCGATGACCATGATTACGCCAAGCTCGCG	
ps4[1]	aTTCCGGGGATCCGTCGACC	Amplifying fragments surrounded by FRT sites
ps1[1]	atGTGTAGGCTGGAGCTGCTTC	
**Northern probes**		
Bs NagA 14 NB	CGGCAAATGGTGAGAATCACCTCGCAATCACTGCTGAC	*nagA*
Bs NagP 15 NB	CATCCACCAAAGCGGTATCCTTCACAGTCAGTCG	*nagP*
GamA 11 NB	GCTGGTACATCTGTCGTGACAGGACCTTCAGCCATC	*gamA*
GamP 12 NB	CGCCTTGTTTGACGGCTTGTCCGCTTGTGACATGTG	*gamP*
PtsG 5 NB	CAGTCACACGCAGACGAGTGATACAAGCATCAAGG	*ptsG*
YbgA 1 NB	CGCAGGCGCTCCGACATTGGTTAATAAGTGTCCGCTG	*ybgA*
YvoA 1 NB	TTCTTCCGTGGTGGCAGCGCTTGGCTCAAGCTC	*yvoA(nagR)*
HP246	ATCGGCGCTGAAGAGCTTAACTTCC	5S RNA
**Primer extension**		
BsNagA 13 RT	GTCATTGATTCCGACATAGCCG	*nagA*
NagP 17 RT	TGCAGGATCCGATGGCAATACCGATCGC	*nagP*
HprK 1 RT	GCACAGAGAATCCATTCGCTGC	*hprK*
GamA 10 RT	ATACAAGCCGACCGGTGTGC	*gamA*
YbgA 2 RT	CATTCGCCTGATATTTGCCCG	*ybgA*

Restriction sites introduced into oligonucleotides are underlined

[1] A and AT were introduced at the 5' end of ps1 and ps4 (Datsenko KA, Wanner BL (2000) Proc Natl Acad Sci USA 97: 6640-6645) to ensure that the scar sequence after elimination of the *spec* cassette forms an in-phase peptide with the start and stop codons of the replaced gene.
