# Supplementary material for: The Use of Amino Sugars by Bacillus subtilis: Presence of a Unique Operon for the Catabolism of Glucosamine
Source: PLoS One. 2013 May 8;8(5):e63025. doi: 10.1371/journal.pone.0063025 (PMC3648570; doi:10.1371/journal.pone.0063025)
Supplement: Table S3 — Doubling times of the gam and nag mutants of B. subtilis during growth on GlcN and GlcNAc. Growth rates are given as doubling times (min) of the different nag and gam mutants during growth on GlcN and GlcNAc. The alternative name for yvoA is nagR [9]. DT were calculated by regression analysis of log OD versus time, generally in range 0.1–1.5 OD. Values are the mean doubling times (± standard deviation) of two to four cultures. NG = no growth. These are the data used to make Figure 5 and mutant strains are numbered as in that figure. (PDF) [file pone.0063025.s005.pdf]

**Table S3. Doubling times of the *gam* and *nag* mutants of *B. subtilis* during growth on GlcN and GlcNAc**

| | | **GlcN** | **GlcNAc** |
|---|---|---|---|
| | *1 wt* | 48.5 ± 0.5 | 93.2 ± 3.6 |
| Enzymes | *2 nagA* | 47.8 ± 1 | NG |
| | *3 gamA* | 479 ± 23 | 190 ± 36 |
| | *4 nagB* | 49.2 ± 1.5 | 97.3 ±3 .4 |
| | *5 gamA nagB* | NG | NG |
| | *6 gamAP* | 322 ± 8 | 225 ± 17 |
| Transporters | *7 gamP* | 51.5 ± 1.9 | 107 ± 9.7 |
| | *8 nagP* | 47.7 ± 1 | 565 ± 56 |
| | *9 gamP nagP* | 51.1 ± 1.1 | 642 ± 110 |
| | *10 ptsG* | 47.7 ± 1 | 108 ± 9 |
| | *11 gamP ptsG* | 135 ± 3 | 142 ± 10 |
| | *12 gamP nagP ptsG* | 140 ± 11 | 593 ± 169 |
| | *13 ypqE* | 48.3 ± 1 | 98.5 ± 7.7 |
| | *14 gamP ptsG ypqE* | 122 ± 6 | 178 ± 21 |
| Regulators | *15 yvoA* | 48.7 ± 1 | 79.9 ± 3.9 |
| | *16 ybgA* | 49.0 ± 0.3 | 74.0 ± 2.2 |
| | *17 yvoA ybgA* | 49.0 ± 1.7 | 71.6 ± 4.0 |
| | *18 yvoA gamP* | 50.6 ± 0.6 | 95.5 ± 8.5 |
| | *19 ybgA nagP* | 49.9 ± 1.6 | 193 ± 38 |
| | *20 yvoA gamA* | 172 ± 23 | 166 ± 16 |
| | *21 yvoA gamAP* | 176 ± 25 | 164 ± 8 |

Growth rates are given as doubling times (min) of the different *nag* and *gam* mutants during growth on GlcN and GlcNAc. The alternative name for *yvoA* is *nagR* [9]. DT were calculated by regression analysis of log OD versus time, generally in range 0.1-1.5 OD. Values are the mean doubling times (± standard deviation) of two to four cultures.

NG =no growth. These are the data used to make Figure 5 and mutant strains are numbered as in that figure.
